# Metabolic engineering of *Escherichia coli* for direct production of vitamin C from D-glucose

**DOI:** 10.1186/s13068-022-02184-0

**Published:** 2022-08-22

**Authors:** Yong-Sheng Tian, Yong-Dong Deng, Wen-Hui Zhang, Yu-Wang, Jing Xu, Jian-Jie Gao, Bo-Wang, Xiao-Yan Fu, Hong-Juan Han, Zhen-Jun Li, Li-Juan Wang, Ri-He Peng, Quan-Hong Yao

**Affiliations:** 1 Shanghai Key Laboratory of Agricultural Genetics and Breeding, Biotechnology Research Institute of Shanghai Academy of Agricultural Sciences, 2901 Beidi Road, Shanghai, People’s Republic of China; 2 Key Laboratory for Safety Assessment (Environment) of Agricultural Genetically Modified Organisms, Ministry of Agriculture and Rural Affairs, Shanghai, China

**Keywords:** Vitamin C, One-step fermentation, D-Glucose, Synthesis pathway

## Abstract

**Background:**

Production of vitamin C has been traditionally based on the Reichstein process and the two-step process. However, the two processes share a common disadvantage: vitamin C cannot be directly synthesized from D-glucose. Therefore, significant effort has been made to develop a one-step vitamin C fermentation process. While, 2-KLG, not vitamin C, is synthesized from nearly all current one-step fermentation processes. Vitamin C is naturally synthesized from glucose in *Arabidopsis thaliana* via a ten-step reaction pathway that is encoded by ten genes. The main objective of this study was to directly produce vitamin C from D-glucose in *Escherichia coli* by expression of the genes from the *A. thaliana* vitamin C biosynthetic pathway.

**Results:**

Therefore, the ten genes of whole vitamin C synthesis pathway of *A. thaliana* were chemically synthesized, and an engineered strain harboring these genes was constructed in this study. The direct production of vitamin C from D-glucose based on one-step fermentation was achieved using this engineered strain and at least 1.53 mg/L vitamin C was produced in shaking flasks.

**Conclusions:**

The study demonstrates the feasibility of one-step fermentation for the production of vitamin C from D-glucose. Importantly, the one-step process has significant advantages compared with the currently used fermentation process: it can save multiple physical and chemical steps needed to convert D-glucose to D-sorbitol; it also does not involve the associated down-streaming steps required to convert 2-KLG into vitamin C.

**Supplementary Information:**

The online version contains supplementary material available at 10.1186/s13068-022-02184-0.

## Introduction

Vitamin C, also known as L-ascorbic acid (L-AA), is a water-soluble vitamin present in biological tissues. Vitamin C is an essential nutrient element that cannot be synthesized by humans and other primates [1]. Vitamin C is widely used in pharmaceuticals, foods, beverages, cosmetics, and animal feed industries [2, 3]. Given the application of vitamin C in different fields, the market demand for vitamin C continues to grow.

The commercial vitamin C is conventionally produced by the Reichstein process [4]. Vitamin C is finally produced through the esterification of L-sorbose into 2-keto-L-gulonic acid (2-KLG) that was obtained using D-glucose as substrate by five chemical reactions and a one-step biological reaction. The process has been the main approach in producing vitamin C for over 60 years, while it has inherent disadvantages of a long production time, complex procedures, high temperature and pressure requirements, and the continuous operation difficulties. In addition, the process requires the use of a large number of toxic chemicals that consume high amounts of energy and that may seriously pollute the environment [5]. Therefore, researchers have tried to find new methods by which to improve the performance of the traditional Reichstein process [5, 6].

Subsequently, a two-step fermentation method that is simpler and more environmentally friendly than Reichstein process was established [7]. However, the direct conversion of D-glucose into vitamin C cannot be achieved. Moreover, three species of bacteria are required for the two-step fermentation, making the process cumbersome. Therefore, novel biotechnological processes that directly convert D-glucose to vitamin C through one-step fermentation would be desirable. Thus, significant effort has been made to develop a one-step vitamin C fermentation process [5]. However, 2-KLG, not vitamin C, is synthesized in almost all current one-step vitamin C fermentation processes [8–13].

**Fig. 1:**
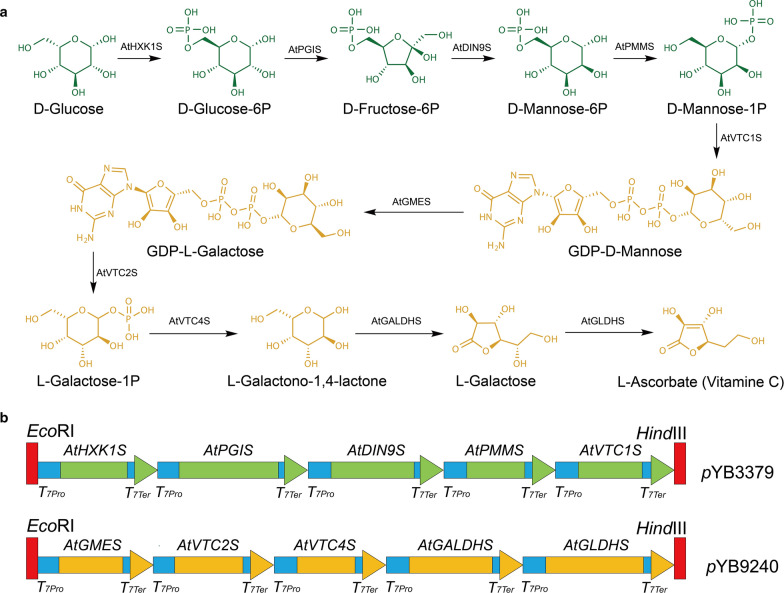
Vitamin C biosynthetic pathway (**a**) and the schematic representation of recombinant vectors (**b**) used in this study

Previous studies have reported that vitamin C biosynthesis exists in most plant species [10, 13]. The biosynthetic pathway of vitamin C in plants was originally proposed based on biosynthesis in animals. However, plant cells do not contain GUL oxidase and do not invert the D-glucose carbon skeleton during its conversion into vitamin C. The specific synthesis pathway of vitamin C in plants was not proposed until mutants (*vtc4-1* and *vtc*) of *A. thaliana* affecting vitamin C biosynthesis were characterized [14, 15]. At present, vitamin C is thought to be synthesized predominantly through the Smirnoff–Wheeler pathway (Fig. 1a). Although some such as *Citrus* sp. and Kiwifruit (*Actinidia chinensis*) are famous for their high content of vitamin C [16], the vitamin C biosynthetic pathway in *A. thaliana* has been well-established. The genes responsible for vitamin C biosynthesis in *A. thaliana* from glucose are *AtHXK1*, *AtPGI*, *AtDIN9*, *AtPMM*, *AtVTC1*, *AtGME*, *AtVTC2*, *AtVTC4*, *AtGalDH*, and *AtGLDH*. Moreover, all of these genes have been studied, and their functions have been established in plants, *Kluyveromyces lactis*, and *Saccharomyces cerevisiae* [10, 17–19]. Therefore, in principle, this has made the direct production of vitamin C from D-glucose achievable. However, to date, there are little reports concerning the direct production of vitamin C from D-glucose by engineering the ten genes involved in the vitamin C biosynthetic pathway of *A. thaliana*.

In this study, the ten genes involved in vitamin C biosynthesis in *A. thaliana* were chemically synthesized, and an engineered *E. coli* strain harboring the ten genes was constructed. The direct production of vitamin C from D-glucose based on one-step fermentation was achieved using this engineered strain.

## Results and discussion

### Biosynthesis of vitamin C from D-glucose in engineered strain B539

Most commercial vitamin C is produced based on the Reichstein process and the two-step fermentation process, while there are inherent economical and environmental disadvantages associated with these processes. One of the most important disadvantages is the higher manufacturing cost of the fermentation process compared to other similar large block fermentation products. If vitamin C could be directly produced from inexpensive D-glucose by a one-step fermentation process, the final cost of vitamin C would be likely to significantly decreased [5]. Theoretically, secondary metabolite compounds can be biosynthesized by the metabolic engineering of genes involved in their biosynthesis pathways in a microbial host. Metabolic engineering of *Pichia pastoris* for the production of riboflavin by coexpression of five genes in the riboflavin synthetic pathway from *B. subtilis* and metabolic engineering of *E. coli* for de novo biosynthesis of vitamin B12 by heterologous expression of a total of 28 genes are typical examples of microbial biosynthesis of secondary metabolite compounds [20, 21]. However, it was unclear whether the heterologous expression of the ten genes in the vitamin C biosynthesis pathway in *E. coli* could achieve the direct production of vitamin C from D-glucose based on a one-step process.

Thus, the main objective of this study was to directly produce vitamin C from D-glucose in *E. coli* by the expression of the genes from the *A. thaliana* vitamin C biosynthetic pathway. A recent study reported that an engineered *K. lactis* strain harboring GDP-mannose 3,5-epimerase (*AtGME*), GDP-L-galactose phosphorylase (*AtVTC2*) and L-galactose-1-phosphate phosphatase (*AtVTC4*) from *A. thaliana* was capable of biosynthesizing vitamin C [22]. Therefore, we first constructed a recombinant vector pYB3379 harboring the last five genes (*AtGMES*, *AtVTC2S*, *AtVTC4S*, *AtGalDHS* and *AtGLDHS*) from the *A. thaliana* vitamin C pathway (Fig. 1b). Several successful experiments have been conducted in *E. coli* multigene engineering in recent years [23, 24]. The examination of these processes reveals that simultaneous coordinated expression of multiple genes in a host background is essential in multigene engineering. Traditional multigene construction approaches often yielded gene expression increases with the distance between the start of a gene and the end of the operon (“transcription distance”) due to increased translation [25]. The multi-monocistronic approaches are capable of expressing multiple genes simultaneously with coordinated control in one vector in which each gene may contain its own promoter and terminator [26]. Hence, the T7 promoter and terminator were selected for controlling the expression of each gene in this study.

Then, the recombinant plasmid pYB3379 was transformed into *E. coli* strain BL21(DE3) to obtain the engineered strain B3379. In addition, shaking flask fermentation of B3379 was carried out to detect whether vitamin C biosynthesis had occurred using UPLC–MS/MS analysis. Vitamin C (precursor ions: m/z175; daughter ions: m/z 115 and 87) was not detected in supernatants from B3379 (see Additional file 1: Figure S1, S2 and S3). Upstream pathways supplying vitamin C precursors may influence vitamin C accumulation, and downstream pathways that metabolize or sequester vitamin C and deplete the vitamin C pool can also drive the reaction forward [23]. GDP-D-mannose, a sugar naturally produced in yeasts for cell wall construction, performs different functions in the synthesis of complex structural carbohydrates between prokaryotes and eukaryotes [14]. Thus, a possible reason behind vitamin C not being detected in the supernatant from B3379 is that a lot of GDP-D-mannose was consumed in the synthesis of complex structural carbohydrates in B3379. This resulted in the metabolic flux did not flow to the vitamin C biosynthesis. If so, vitamin C biosynthesis could be achieved by improving the supply of GDP-D-mannose and generating a metabolic sink to store the products.

**Fig. 2:**
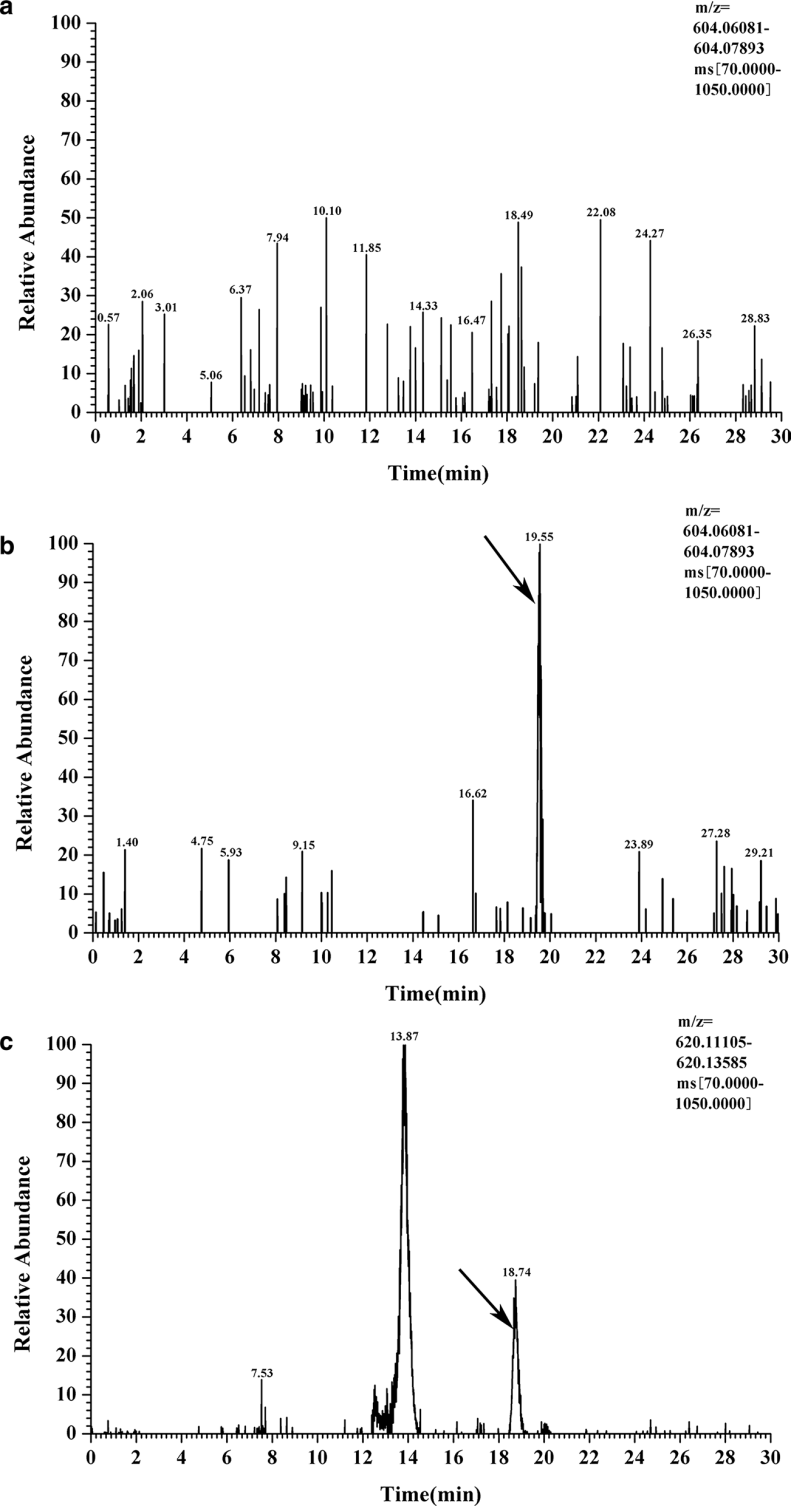
HPIC–QE–MS analysis of [U–^13^C]–GDP-D-mannose from different strains. **a** GDP-D-mannose in B5702; **b** GDP-D-mannose in B9240; **c** [U–^13^C]–GDP-D-mannose in B9240

**Fig. 3:**
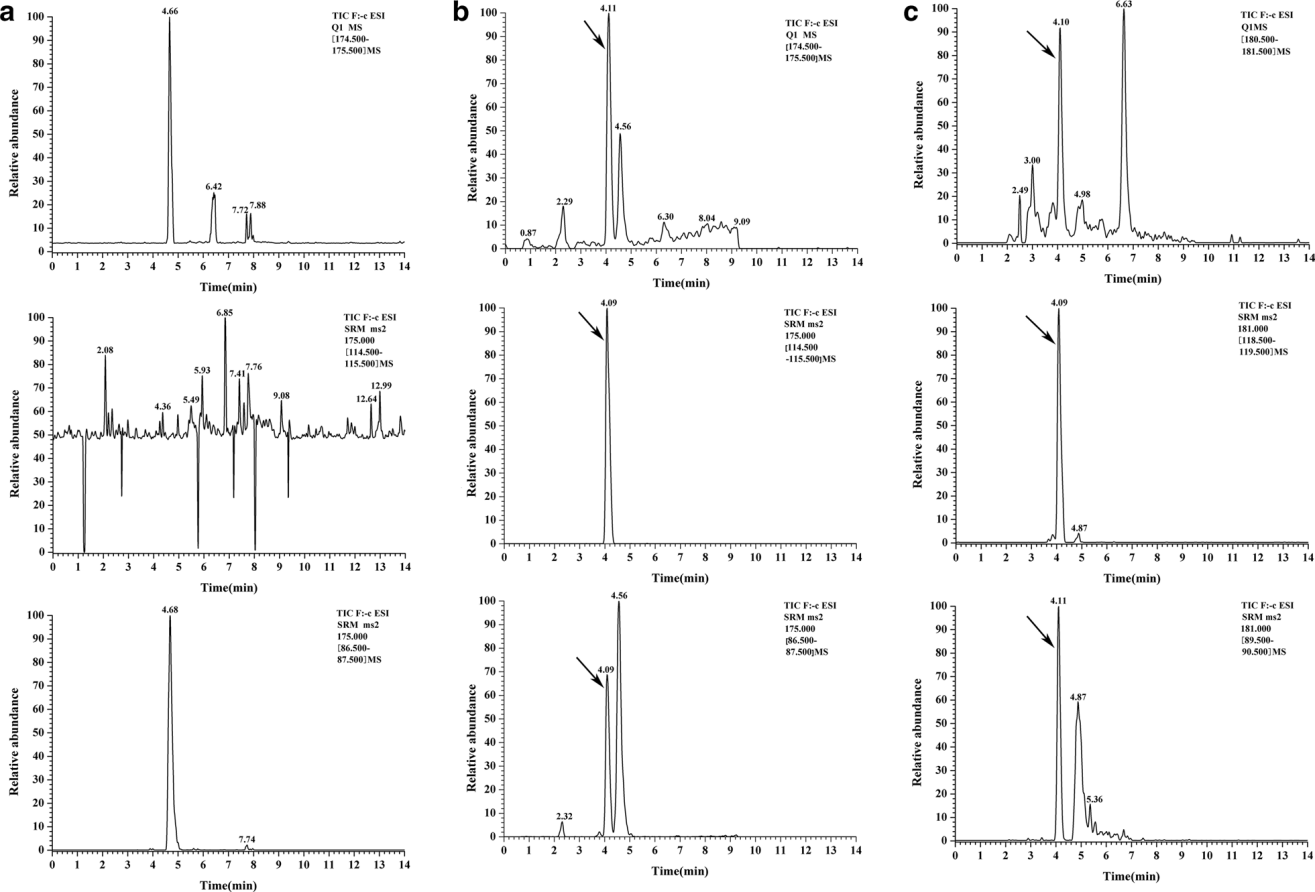
UPLC–MS/MS SIM chromatogram analysis of vitamin C from strain B5703 (**a**) and B539 (**b**) at 8 h induction with arabinose; UPLC–MS/MS SIM chromatogram analysis of U–^13^C-vitamin C from strain B539 (**c**) at 8 h induction with arabinose (upper: M/Z175 or M/Z 181; middle: M/Z119 or M/Z 115; bottom: M/Z90 or M/Z 87)

To this end, a recombinant vector pYB9240 carrying *A. thaliana* genes for the GDP-D-mannose biosynthetic pathway was constructed using the same method and transformed into *E. coli* BL21 (DE3), generating strain B9240 (Fig. 1b). Then, shaking flask fermentation of B9240 was carried out to detect whether there was GDP-D-mannose biosynthesis by HPIC–QE–MS analysis. As shown in Fig. 2, GDP-D-mannose was detected in supernatants of B9240 and not in the control strain B5702 [the *E. coli* BL21 (DE3) transformed with vector pBEPMOB]. This result suggested that the engineered strain B9240 could synthesize GDP-D-mannose. To achieve vitamin C synthesis, pYB9240 was transformed into *E. coli* BL21 (DE3) alongside plasmid pYB3379, generating strain B539. Then, UPLC–MS/MS analysis of supernatants from B539 was performed to verify the vitamin C biosynthesis. As expected, vitamin C (precursor ions: m/z 175; daughter ions: m/z 115 and 87) was detected in supernatants from B539 but not in the control strain B5703 [the *E. coli* BL21 (DE3) transformed with two empty vectors (pBR326 and pBEPMOB)] (Fig. 3a and b). To further demonstrate that the biosynthesis of vitamin C was derived from D-glucose, [U–^13^C]-glucose was used as a substrate and added into the M9 medium for shaking flask fermentation. Similarly, [U–^13^C]-vitamin C (precursor ions: m/z 181; daughter ions: m/z 119 and 90) was detected in supernatants from B539 (Fig. 3c). The [U–^13^C]–GDP-D-mannose was also detected in supernatants from B539 (Fig. 2c). The results suggested that a supply of GDP-D-mannose from upstream is one of the key factors limiting downstream vitamin C accumulation. The results also demonstrated that the introduction of the *A. thaliana* vitamin C biosynthetic pathway enables de novo direct biosynthesis of vitamin C (1.53 mg/L) from D-glucose in *E. coli* based on a one-step process.

### Expression of ten genes in engineered strain B539

**Fig. 4:**
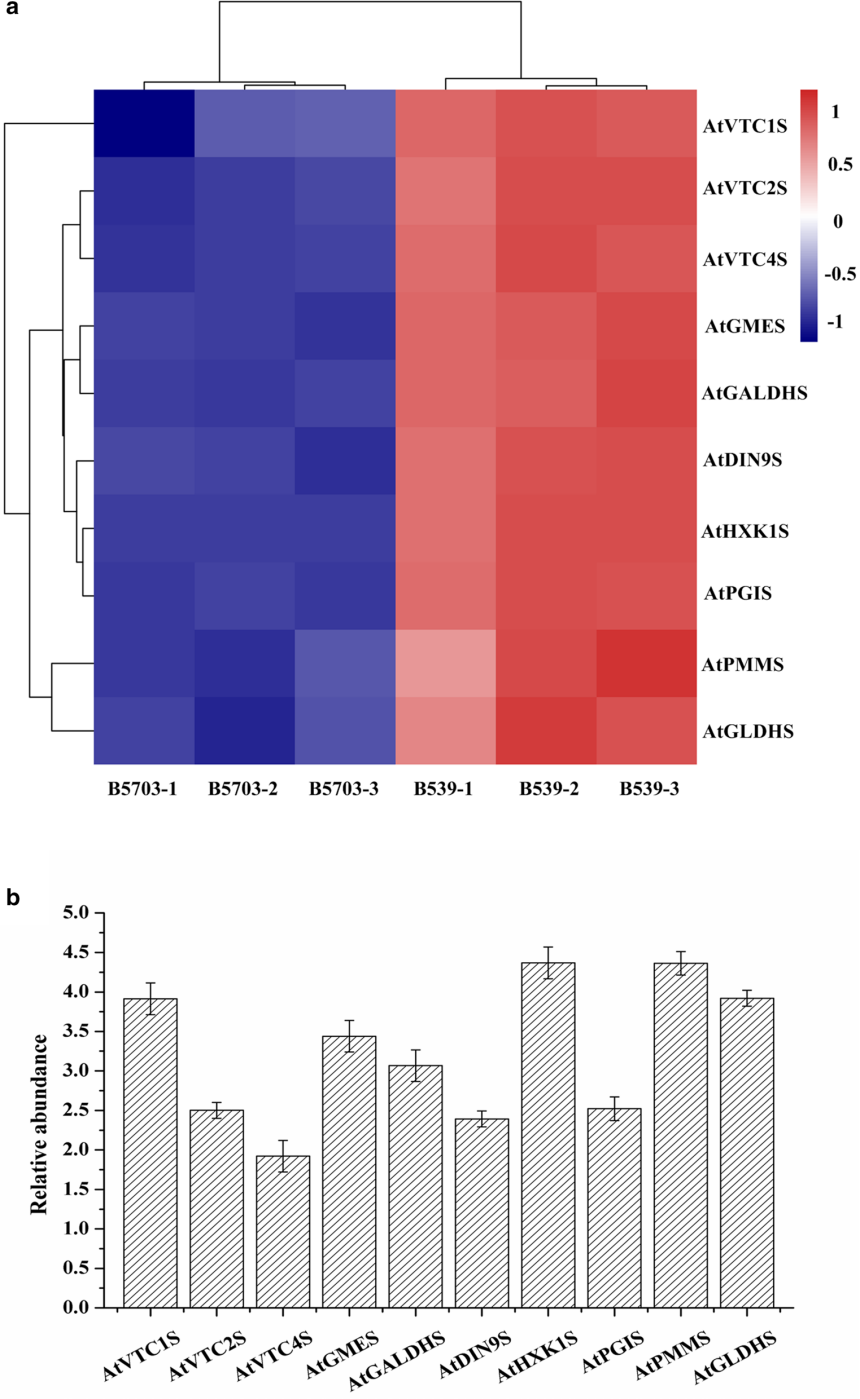
Protein expression analysis; a, heatmap of relative protein expression difference comparison between B539 and B5703 (the deeper the red color, the higher the expression, and the deeper the blue the color, the lower the expression); b, comparation of relative protein abundance among ten proteins in B539

The development of metabolic engineering by multigene transformation has seen increasingly engineered pathways composed of two to three enzymes initially progressing to pathways containing ten or more enzymes at present [23]. The more enzymatic steps in a heterologous pathway, the more challenging the construction of the pathway will be because of cloning difficulties, stability issues, and the declining efficiency of transformation of ever-larger DNA fragments. In addition, the vector carrying multiple genes will become increasingly cumbersome and unstable as the number of transgenes increases [27]. Therefore, the successful construction of the expression vector must first be confirmed by polymerase chain reaction (PCR) using plasmid isolated from the transformant. All ten transgenes were detected in transformants by PCR in this study (see Additional file 1: Table 1 and Figure S4). The results indicated that the vector with ten genes was stable, and all ten genes were completely expressed. Then, to ensure the adequate expression of functional enzymes in the transformants, the transcription levels of the ten genes were analyzed through qPCR using ten pairs of specific primers (see Additional file 1: Table S2). Although there were minor differences in transcription levels among the ten transgenes, all of the transgenes were overexpressed in the transformants (see Additional file 1: Figure S5). The results demonstrated that the genes were actively and stably transcribed in transformants. Previous studies have reported that an enzyme may function suboptimally or not at all after the introduction of multiple genes into a heterologous host, and this may result in bottlenecks of carbon flux from upstream metabolism into downstream pathways [23]. Therefore, proteomic assays were conducted to evaluate the expression of the proteins produced by the ten genes. The results suggested that the individual proteins of the ten genes were correctly expressed (Fig. 4). Although there were minor differences in protein expression among the ten proteins, the result of qualitative detection of vitamin C (Fig. 3) demonstrated that these proteins at least performed their respective functions.

**Table 1:** Metabolic flux

List of metabolite	Strains	Times (induction with arabinose)				
		0 h	2 h	4 h	6 h	8 h
D-Glucose-6P	B539	728,589	42,041,950	12,357,204	5,541,360	3,718,343
	B5703	1,137,825	35,135,366	16,812,928	7,763,633	4,848,915
D-Fructose-1,6-2P	B539	0	111,014,765	6,182,002	894,153	612,773
	B5703	942,182	39,481,007	3,851,501	299,258	114,375
Ribose-5P	B539	0	1,505,483	2,223,421	273,274	243,755
	B5703	0	1,474,348	1,611,914	512,025	558,143
PEP	B539	126,006	22,731,977	3,331,449	2,434,459	3,248,891
	B5703	380,990	1,931,877	5,752,892	5,219,953	4,746,976
Pyruvate	B539	398,717	84,632,477	58,309,713	49,395,212	77,246,204
	B5703	460,026	73,558,546	57,521,203	59,415,845	81,803,110
Citrate	B539	169,209	87,009,353	3,854,470	19,968,252	26,807,885
	B5703	674,282	61,961,209	8,331,791	20,782,970	31,364,602
a-Ketoglutarate	B539	93,545	904,946	4,033,714	7,213,857	20,397,767
	B5703	38,816	598,282	7,603,855	16,456,613	24,207,181
Fumarate	B539	4,677,244	8,186,085	4,263,780	3,333,971	3,158,021
	B5703	2,103,875	3,600,110	7,220,976	5,007,847	4,278,011
Malate	B539	2,445,713	19,993,286	1,830,362	1,204,770	2,089,227
	B5703	4,241,743	9,294,525	2,059,973	1,493,029	2,437,944
D-Mannose-6P	B539	0	14,693	29,847	0	0
	B5703	0	0	0	0	0
GDP-D-Mannose	B539	0	0	9920	42,742	20,012
	B5703	0	0	0	0	0
L-Galactono-1,4-lactone	B539	1515	162,695	294,489	386,450	316,948
	B5703	3474	11,265	16,427	20,147	25,899
D-Galacturonate	B539	0	106,514	117,279	86,389	202,064
	B5703	0	326,280	170,989	187,455	257,359

The values are the peak areas (relative content). The values at 0 h are the relative content of the unlabeled metabolite; the values at 2 h, 4 h, 6 h, and 8 h are the relative contents of ^13^C-labeled metabolites

### Analysis of residual sugar, cell dry weight and the production of vitamin C in engineered strain B539

**Fig. 5:**
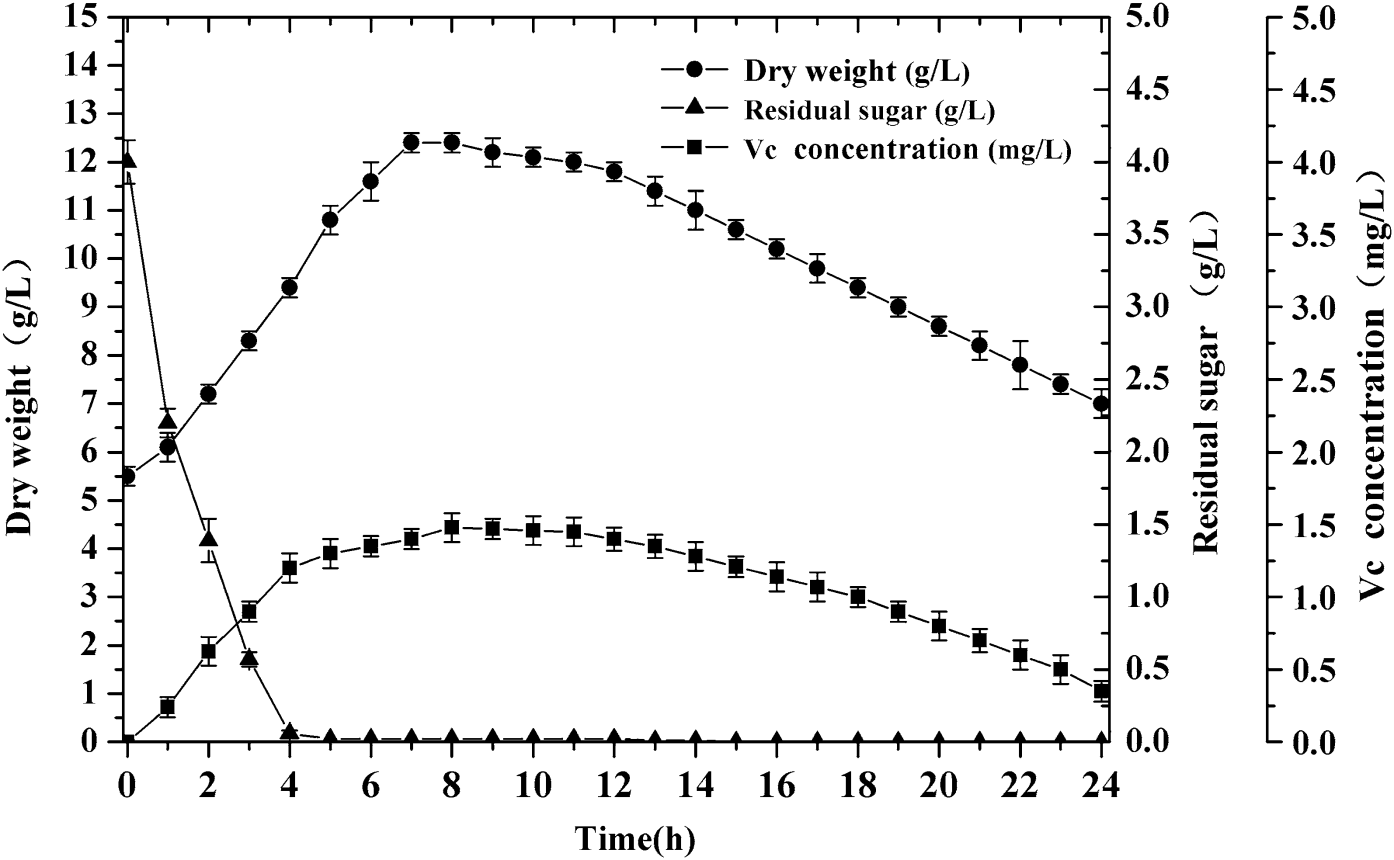
Changes in vitamin C production, cell dry weight, and residual sugar from strain B539 during 24 h induction with arabinose

After gene transfer and coordinated expression in a microorganism background, the ultimate goal is to achieve the accumulation of target compounds. Figure 5 shows the changes in the main indexes of strain B539 during 0–24 h induction with arabinose. As shown in Fig. 5, the glucose was quickly consumed, and the content of residual sugar was nearly zero at 4 h due to glucose acting not only as a carbon source but also as a substrate. Similarly, the dry weight of cells also showed a trend of first increasing and then decreasing, reaching the maximum dry weight of 12.4 g/L at 7 h and then declining to the minimum dry weight of 7.0 g/L at 24 h. The production of vitamin C gradually increased from 0 to 1.53 mg/L during 8 h induction with arabinose. Then, the produced vitamin C gradually decreased with time and could not be detected after 24 h. A possible reason for this result is that the ventilation fermentation process may destroy most of the produced vitamin C due to its instability and susceptibility to oxidation [5]. Another possible reason is based on the fact that most D-glucose is rapidly consumed as a carbon source at the early stage of fermentation to maintain the normal vital activities of the strain, resulting in almost no D-glucose acting as a substrate to drive the metabolic flux flow to vitamin C production during the middle and later stages of fermentation. These results demonstrated that the engineered strain B539 harboring *A. thaliana* vitamin C biosynthetic pathway can directly produce vitamin C from D-glucose.

### Targeted metabolomics

**Fig. 6:**
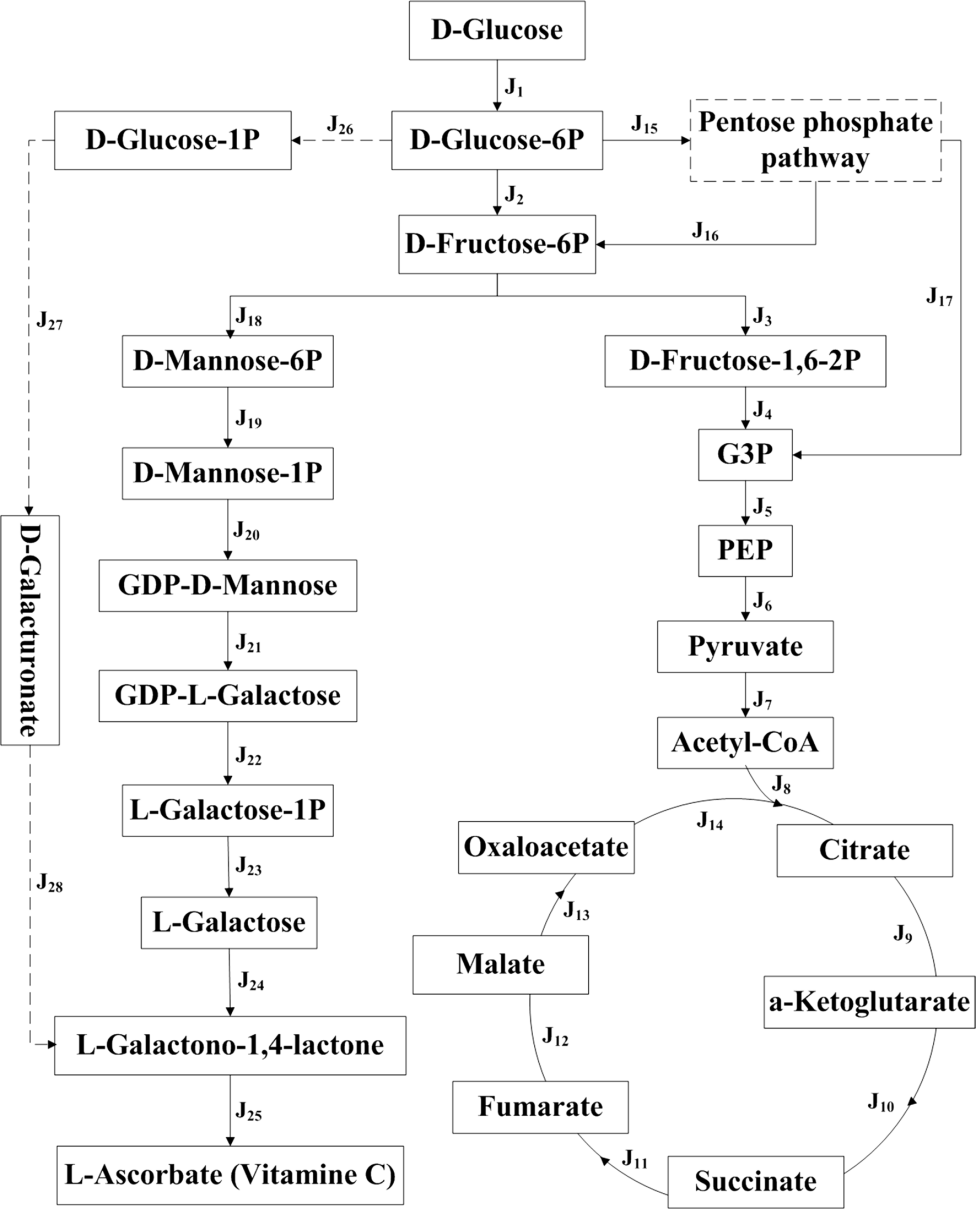
Metabolic networks

D-glucose can be used as both a carbon source and a substrate for the synthesis of vitamin C; thus, the metabolic pathway of D-glucose in strain B539 is more complex than in the control strain B5703. Therefore, [U–^13^C]-glucose was used to conduct a comparative analysis of the targeted metabolomics of the two strains by detecting changes in metabolites in the main metabolic pathways [including the glycolytic pathway (EMP), the pentose phosphate pathway (PPP), the tricarboxylic acid (TCA) cycle pathway, and the constructed vitamin C synthesis pathway] at various times (0 h, 2 h, 4 h, 6 h, and 8 h). As shown in Fig. 6, the metabolic pathways from D-glucose to fructose-6P in B539 and B5703 were the same. However, for B539, the subsequent flow of fructose-6P was divided into two paths, one to fructose-1,6-2P (J_3_) that enters the glycolysis pathway to maintain the normal vital activities of the bacteria, and another that leads to mannose-6P (J_18_) and to finally synthesize vitamin C after seven additional reaction steps. As shown in Table 1, the key intermediate metabolites (mannose-6P, GDP-D-mannose, and L-galactono-1,4-lactone) in the synthesis pathway of vitamin C were detected in the fermentation broth of strain B539. The concentration of mannose-6P gradually increased from 0 to 4 h, after which it was gradually consumed. GDP–D-mannose was detected at 4 h, and its concentration gradually increased from 4 to 6 h, then gradually decreased from 6 to 8 h. Interestingly, L-galactono-1,4-lactone was detected in the fermentation broths of both B539 and B5703. Metabolic network analysis to determine the source of L-galactono-1,4-lactone in strain B5703 revealed the possible synthesis pathway [28] (see Additional file 1: Figure S6). Importantly, the metabolite D-galacturonate was also detected in the fermentation broth (Table 1), further supporting this speculation. Although strain B5703 could produce L-galactono-1,4-lactone through its metabolism, the L-galactono-1,4-lactone content differed significantly in the fermentation broth of strains B539 and B5703. The content of L-galactono-1,4-lactone in the fermentation broth of strain B539 was 14.4 times higher at 2 h and 17.9 times higher at 4 h than in the fermentation broth of strain B5703. The content of L-galactono-1,4-lactone in strain B539 showed rapid accumulation between 0 and 6 h, then decreased from 6 to 8 h. These findings indicated that L-galactono-1,4-lactone was used as a precursor to gradually produce vitamin C. However, the L-galactono-1,4-lactone in the fermentation broth of strain B5703 constantly accumulated, indicating that the production of L-galactono-1,4-lactone mainly originated from the hypothesized bypass pathway (J_26_–J_28_). Most importantly, L-galactono-1,4-lactone in the fermentation broth of strain B539 was considerably higher than that in strain B5703, suggesting that the biosynthesis of vitamin C occurred along the metabolic pathway we constructed, further confirming that this biosynthesis was based on a one-step process to directly biosynthesize vitamin C using D-glucose as a substrate.

The results also revealed that the accumulation rate of the metabolite D-glucose-6P in the fermentation broth of strain B539 was 1.2 times higher than that of B5703 during 0–2 h, and the consumption rate of D-glucose-6P during 2–8 h was also higher than in strain B5703 (Table 1). For example, the content of D-glucose-6P of strain B539 decreased by 70% from 2 to 4 h, while in strain B5703 this only decreased by 52%. These findings indicate that although *E. coli* has an inherent metabolic pathway from glucose to D-glucose-6P, the introduction of the exogenous genes *AtHXK1S* and *AtPGIS* into strain B539 allowed the metabolic efficiency of this pathway to increase. This in turn helped to synthesize the intermediate metabolites faster to further synthesize the target metabolite vitamin C. Similarly, the accumulation rates of D-fructose-1,6-2P, phosphoenol pyruvic acid, pyruvic acid, and the organic acids (citrate, a-ketoglutarate, fumarate, and malate) in the TCA cycle in the fermentation broth of strain B539 within 0–2 h were also substantially higher than in B5703, and this was because the introduction of the exogenous genes *AtHXK1S* and *AtPGIS* accelerated the upstream metabolic efficiency. However, the amounts of these substances that accumulated in the fermentation broth of strain B5703 gradually exceeded those of B539 after 2 h (Table 1). This occurred, because part of the fructose-6P in strain B539 was used as a substrate to synthesize vitamin C through the seven-step reaction; thus, the contents of some key metabolites of EMP became gradually lower than those of strain B5703. The changes of key metabolites further confirmed the feasibility of a one-step fermentation process to synthesize vitamin C in *E. coli.*

## Conclusions

The whole vitamin C synthesis pathway of *A. thaliana* was introduced into *E. coli* and the direct production of vitamin C from D-glucose based on one-step fermentation was achieved. The engineered strain was capable to produce at least 1.53 mg/L of vitamin C at 8 h of induction. Although the yields of vitamin C from this engineering strain is not yet competitive with others engineered strain [29], the engineered strain in this study has a doubling time that is 3–4 times shorter than others engineered strain, and is well-suited to very high expression of enzymes [30]. Importantly, the one-step process has significant advantages compared with the currently used fermentation process: it can save multiple physical and chemical steps needed to convert D-glucose to D-sorbitol; it also does not involve the associated down-streaming steps required to convert 2-KLG into vitamin C. Thus, the one-step process involving this engineered strain will provide an alternative option for simple production of vitamin C in the future.

## Materials and methods

### Synthetic target genes and vector construction

The ten genes of the vitamin C biosynthesis pathway from *A. thaliana* were codon-optimized and chemically synthesized through PCR-based two-step DNA synthesis (PTDS) strategies [31, 32]. According to *E. coli* codon usage, the synthetic genes were named *AtHXK1S*, *AtPGIS*, *AtDIN9S*, *AtPMMS*, *AtVTC1S*, *AtGMES*, *AtVTC2S*, *AtVTC4S*, *AtGalDHS*, and *AtGLDHS*. Then, a gene expression cassette was constructed by connecting each gene’s ORF between a T7 promoter and a T7 terminator through PAGE-mediated overlap extension PCR [33]. The first five gene (*AtHXK1S*, *AtPGIS*, *AtDIN9S*, *AtPMMS* and *AtVTC1S*) expression cassettes were re-amplified with primers containing restriction enzyme digestion sites. Then, the PCR fragment was digested using restriction endonucleases and inserted into a pBR326 vector digested with same restriction endonucleases (Genbank No. OK052603) in proper order, yielding pBR326–*AtHXK1S*–*AtPGIS*–*AtDIN9S*–*AtPMMS*–*AtVTC1S* (pYB9240) (see Additional file 1: Table S3). The last five gene (*AtGMES*, *AtVTC2S*, *AtVTC4S*, *AtGalDHS* and *AtGLDHS*) expression cassettes were re-amplified with primers containing restriction enzyme digestion sites. Then, the PCR fragment was digested using restriction endonucleases and inserted into a pBEPMOB vector (Genbank No. OL333432) in proper order, yielding pBEPMOB–*AtGMES*–*AtVTC2S*–*AtVTC4S*–*AtGalDHS*–*AtGLDHS* (pYB3379) (Fig. 1b).

### Bacterial cultures and shaking-flask fermentation

The final construct was transformed into *E. coli* BL21 (DE3) and plated on 2YT agar plates supplemented with ampicillin (pYB9240), kanamycin (pYB3379), or combined ampicillin–kanamycin (pYB9240 and pYB3379). Single colonies after overnight incubation at 37 °C were inoculated into 10 mL LB medium supplemented with 10 g/L glycerin. Then, the cells were inoculated into 100 mL M9 medium supplemented with 10 g/L glycerin, 1 g/L casamino acids, and 10 ppm thiamine hydrochloride and cultured at 37 °C until the optical density at 600 nm (OD600) reached 0.6.

Cells were then resuspended in the same M9 medium except for the 10 g/L glycerin, which was replaced by D-glucose until the OD600 reached 2.0. Then, 2 g/L arabinose were added at 30 °C for the simultaneous induction of gene expression. Aliquots were taken at 8 h to perform qualitative detection (vitamin C and GDP-D-mannose) and proteomic analyses. Aliquots were taken at an interval of 1 h during 24 h induction for subsequent experiments, including the quantitative detection of vitamin C biosynthesis, measurement of cell dry weight, and analysis of residual sugar. [U–^13^C]-glucose was added to the bacterial culture in the aforementioned M9 medium, and aliquots were taken at 8 h to perform the qualitative detection of [U–^13^C]-vitamin C and GDP-D-mannose. Further aliquots were taken at 0 h, 2 h, 4 h, 6 h, and 8 h to perform targeted metabolomics analyses. The *E. coli* BL21 (DE3) transformed with two empty vectors (pBR326 and pBEPMOB) was named B5703 and was used as a control for strain B539 under the same conditions. The *E. coli* BL21 (DE3) transformed with vector pBEPMOB was named B5702 and was used as a control strain for B9240 under the same conditions.

### Gene expression analysis

Plasmid was extracted from the transformants after 8 h of induction. The transgenic nature of the transformants was first confirmed by PCR analysis of extracted plasmid using specific primers (see Additional file 1: Table S1). Total RNA was isolated from the transformants after 8 h of induction using the TRIzol reagent according to the manufacturer’s recommendations. Quantitative real-time PCR (qRT-PCR) of the ten genes was performed according to Tian et al. [34]. Primers used in this study are listed in Additional file 1: Table S2. qRT-qPCR was run in a BIO-RAD iCycler IQ Multi-Color Real Time PCR Detection System (Bio-Rad, Hercules, CA, USA).The relative levels of the amplified mRNA were evaluated according to the 2^−ΔΔ^Ct method.

### Protein analysis

Fermentation broth (5 mL) was centrifuged at 12,000 g for 8 min at 4 °C. Then, the supernatants were discarded, and the resulting protein pellet was shock-frozen in liquid nitrogen and stored at − 80 °C. Proteomics assay was used to analyze the protein level of all genes based on previous study (see supplemental material Note 2) [35].

### Determination of vitamin C, residual sugar, and cell dry weight

Qualitative analysis of vitamin C was performed based on retention time and characteristic ions. Quantitative analysis of vitamin C was performed based on the standard curve of peak area versus concentration. Samples of 10 mL of fermentation broth were centrifuged at 12,000 g for 8 min at 4 °C, and the supernatants were freeze-dried using a vacuum freeze dryer. Subsequently, the dried sample was reconstituted with 100 μL of 0.1% (vol/vol) formic acid in water. The samples were transferred into LC vials for ultra-performance liquid chromatography–tandem mass spectrometry (UPLC–MS/MS) analysis. UPLC–MS/MS analysis was performed on a Thermo Vanquish UPLC system equipped with a C18-AQ column (4.6 × 250 mm, SHIMADZU), connected to a Thermo TSQ Quantum Mass Spectrometer. The mass spectrometer was operated in the negative ion and multiple reaction monitoring (MRM) modes. The optimized parameters were as follows: the ion spray voltage was 3500 V, and the values for the nebulizer gas (GS1), auxiliary gas (GS2), curtain gas (CUR), and collision gas (CAD) were 60, 60, 30, and 15 (arbitrary units), respectively. The sample was injected by the autosampler into mobile phase flow (80% A:20% B; A: 0.1 vol % formic acid in water; B: methanol) at a flow rate of 1 mL/min.

Residual sugar was determined using a D-glucose Content Assay Kit (AKSU001C. Boxbio, China).

The cell dry weight was measured based on a previously described method [36].

### Targeted metabolomics

For the metabolomics analysis, 10 mL of fermentation broth was first centrifuged at 12,000 g for 8 min at 4 °C. The supernatants were dried under a gentle stream of nitrogen, and the samples were extracted using 1000 μL of solution (methanol:water = 3:1, vol/vol). Then, samples were vortexed for 60 s, sonicated for 15 min in an ice-water bath, and vibrated at 4 °C for 15 min followed by incubation at − 40 °C for 1 h. The obtained samples were centrifuged at 12,000 g for 15 min at 4 °C. Then, 900 μL of the clear supernatant was collected and dried under a gentle stream of nitrogen. The residue was reconstituted with ultrapure water. The reconstituted samples were vortexed and sonicated for 10 min in an ice water bath before filtration through the centrifuge tube filter, and the supernatants were subsequently transferred to inserts in injection vials for High Performance Ion Chromatography (HPIC)–QE–MS analysis.

### ***HPIC***–***QE***–***MS analysis***

The HPIC separation was carried out using a Thermo Scientific Dionex ICS-6000 HPIC System (Thermo Scientific) equipped with Dionex IonPac AS11-HC (2 × 250 mm) and AG11-HC (2 mm × 50 mm) columns. Mobile phase A was 100 mM NaOH in water, and phase C was water. Another pumping system was used to supply the solvent (2 mM acetic acid in methanol) and the solvent was mixed with effluent before entering the ion source (flow rate of 0.15 mL/min). The column temperature was set at 30 °C. The auto-sampler temperature was set at 4 °C, and the injection volume was 5 μL.

A QE mass spectrometer was used for its ability to acquire MS spectra in full ms mode via the acquisition software (Xcalibur 4.0.27, Thermo). In this mode, the acquisition software continuously evaluated the full scan MS spectrum. The ESI source conditions were set as follows: sheath gas flow rate as 30 Arb, Aux gas flow rate as 10 Arb, capillary temperature 350 °C, full MS resolution as 35,000, and spray Voltage as − 3.8 kV (negative).

The raw data were converted to the mzXML format using ProteoWizard (Massconvert) and were processed for peak detection, extraction, alignment, and integration using an in-house program that was developed using R and XCMS.

## Supplementary Information

**Additional file 1:** **Table S1. **Primers for molecular analysis of transformation using extracted plasmid as template. **Table S2.** Primers for molecular analysis of transformation using cDNA as template. **Table S3. Escherichia coli** strains used in this study. **Figure S1.** UPLC–MS/MS SIM chromatogram analysis of vitamin C from strain B3379 at 8h induction with arabinose (upper: M/Z175; middle: M/Z 115; bottom: M/Z 87). **Figure S2.** UPLC–MS/MS SIM chromatogram analysis of standard vitamin C (upper: M/Z175; middle: M/Z 115; bottom: M/Z 87). **Figure S3. **UPLC–MS/MS SIM chromatogram analysis of standard U-13C-vitamin C (upper: M/Z181; middle: M/Z 119; bottom: M/Z 90). **Figure S4.** Expression of the transgenes in transformant by PCR using DNA as template (1:*AtHXK1S*; 2:*AtPGIS*; 3:*AtDIN9S*; 4:*AtPMMS*; 5:*AtVTC1S*; 6:*AtGMES*; 7:*AtVTC2S*; 8:*AtVTC4S*; 9:*AtGalDHS*; 10:*AtGLDHS*. M: marker). **Figure S5. **Relative expression of the transgenes in transformant B539 by qPCR. Data shown are representative of three independent experiments. **Figure S6.** Presumably synthesis pathway (green) of L-galactono-1,4-lactone in strain B5703 (KEGG map00053: Ascorbate and aldarate metabolis). Notes 1: The sequence information after codon optimization and the primers used for gene synthesize through PCR-based two-step DNA synthesis (PTDS) strategies. Notes 2: Detailed procedures of Proteomics assay.

## Data Availability

All data generated or analysed during this study are included in this published article and its Additional information files.

## Abbreviations

2-KLG: 2-Keto-L-gulonic acid
L-AA: L-ascorbic acid
PTDS: PCR-based two-step DNA synthesis
UPLC–MS/MS: Ultra-performance liquid chromatography–tandem mass spectrometry
MRM: Multiple reaction monitoring
HPIC: High performance ion chromatography
EMP: Glycolytic pathway
PPP: The pentose phosphate pathway
TCA: The tricarboxylic acid
