# Supplementary material for: Metabolic engineering of Escherichia coli for direct production of vitamin C from D-glucose
Source: Biotechnol Biofuels Bioprod. 2022 Aug 22;15:86. doi: 10.1186/s13068-022-02184-0 (PMC9396866; doi:10.1186/s13068-022-02184-0)
Supplement: Supplementary file 1 — Additional file 1: Table S1. Primers for molecular analysis of transformation using extracted plasmid as template. Table S2. Primers for molecular analysis of transformation using cDNA as template. Table S3. Escherichia coli strains used in this study. Figure S1. UPLC–MS/MS SIM chromatogram analysis of vitamin C from strain B3379 at 8h induction with arabinose (upper: M/Z175; middle: M/Z 115; bottom: M/Z 87). Figure S2. UPLC–MS/MS SIM chromatogram analysis of standard vitamin C (upper: M/Z175; middle: M/Z 115; bottom: M/Z 87). Figure S3. UPLC–MS/MS SIM chromatogram analysis of standard U-13C-vitamin C (upper: M/Z181; middle: M/Z 119; bottom: M/Z 90). Figure S4. Expression of the transgenes in transformant by PCR using DNA as template (1:AtHXK1S; 2:AtPGIS; 3:AtDIN9S; 4:AtPMMS; 5:AtVTC1S; 6:AtGMES; 7:AtVTC2S; 8:AtVTC4S; 9:AtGalDHS; 10:AtGLDHS. M: marker). Figure S5. Relative expression of the transgenes in transformant B539 by qPCR. Data shown are representative of three independent experiments. Figure S6. Presumably synthesis pathway (green) of L-galactono-1,4-lactone in strain B5703 (KEGG map00053: Ascorbate and aldarate metabolis). Notes 1: The sequence information after codon optimization and the primers used for gene synthesize through PCR-based two-step DNA synthesis (PTDS) strategies. Notes 2: Detailed procedures of Proteomics assay. [file 13068_2022_2184_MOESM1_ESM.docx]

**Metabolic Engineering of *Escherichia coli* for Direct Production of Vitamin C from D-Glucose**

**Table S1:** Primers for molecular analysis of transformation using extracted plasmid as template

| Gene names | Primer Names | Oligonucleotides  sequences | fragment length  (bp) |
| --- | --- | --- | --- |
| *AtHXK1S* | ATHXK1S-F | CTTGAAGATCGTTGGTTC | 310 |
|  | ATHXK1S-R | GTGAGTAACCTCAACAGA |  |
| *AtPGI1S* | AtPGI1S-F | TTGTACGCATCTATCGTC | 302 |
|  | AtPGI1S-R | TTGCACTCACCGAGGTAG |  |
| *AtDIN9S* | AtDIN9S-F | GGATACCCTGAGATCCTC | 309 |
|  | AtDIN9S-R | CTGGAAGAATCTGGAAGA |  |
| *AtPMMI* | AtPMMS-F | GTAGAAACTGCTCTCAG | 319 |
|  | AtPMMS-R | CACTTTGCAACAGTGTCG |  |
| *AtVTC1S* | AtVTC1S-F | ATCGTTGGAAACGTTCTC | 320 |
|  | AtVTC1S-R | GGCTTGAGGATGTTGGAC |  |
| *AtGMES* | AtGMES-F | ATCTGACGAGATGGTCTC | 315 |
|  | AtGMES-R | CCGTCAGCTGCTCTGAGA |  |
| *AtVTC2S* | AtVTC2S-F | GCTCTCGGAGAAGTTTCT | 312 |
|  | AtVTC2S-R | ACGAGACACTCAGCAGCA |  |
| *AtVTC4S* | AtVTC4S-F | GCTACCTTGGACGACACC | 302 |
|  | AtVTC4S-R | GCTCCAGTGAGTCTGAGT |  |
| *AtGALDHS* | AtGALDHS-F | CTCACCGAACAAGGACCA | 294 |
|  | AtGALDHS-R | GGCCAGGTGAGGTTCTTG |  |
| *AtGLDHS* | AtGLDHS-F | ATCCAAGTCTCCTATCTC | 294 |
|  | AtGLDHS-R | TCAAGCTCTCTTCTTGCC |  |

**Table S2:** Primers for molecular analysis of transformation using cDNA as template

| Gene names | Primer Names | Oligonucleotides sequences |
| --- | --- | --- |
| *AtHXK1S* | ATHXK1S-F | TTGGGTTTCACCTTCTCCTTC |
|  | ATHXK1S-R | TCCTGTCCGACTGCCTCC |
| *AtPGI1S* | AtPGI1S-F | GAGACTACCTCTTCGGAATGCT |
|  | AtPGI1S-R | RGACGATAGATGCGTACAAACCA |
| *AtDIN9S* | AtDIN9S-F | TACGCTGAGTTCTGGATGGG |
|  | AtDIN9S-R | AACCTTGAAGAGGAATGGGAG |
| *AtPMMI* | AtPMMS-F | AACTTCACCCTCCACTACATCG |
|  | AtPMMS-R | CGTTGAGCATTCCGTTTCTG |
| *AtVTC1S* | AtVTC1S-F | CAAAGATCGCAGCAGCACA |
|  | AtVTC1S-R | CAGAGGTCAACTTGGCAGGA |
| *AtGMES* | AtGMES-F | ACTGGAAGAAGAACGAGCACAT |
|  | AtGMES-R | CAGCAGCCAAGTTGAAGACG |
| *AtVTC2S* | AtVTC2S-F | GCTGAGGCTGCTAACCCATA |
|  | AtVTC2S-R | GGAATGGCATAGCGAGGTAG |
| *AtVTC4S* | AtVTC4S-F | TTACCGAACTCACCGACGAA |
|  | AtVTC4S-R | TCAAGAATGCTCCCTTACCCT |
| *AtGALDHS* | AtGALDHS-F | TCTGAGAAGATGCTCGGTAAGG |
|  | AtGALDHS-R | GGACTCGTCGATGGACTTTCT |
| *AtGLDHS* | AtGLDHS-F | AGATGCGGACTTGGTGGACT |
|  | AtGLDHS-R | CAACAACGGTGTCAGTGTATGG |

**Table S3:** *Escherichia coli* strains used in this study

| **Strains** | **Markers** | **Cassette expression** | **Plasmids** |
| --- | --- | --- | --- |
| B9240 | Amp^R^ | *AtHXK1S*, *AtPGIS*, *AtDIN9S*, *AtPMMS*, *AtVTC1S* | pYB9240 |
| B3379 | Kan^R^ | *AtGMES*,*AtVTC2S*,*AtVTC4S*,*AtGalDHS*,*AtGLDHS* | pYB3379 |
| B539 | Amp^R^ Kan^R^ | *AtHXK1S*, *AtPGIS*, *AtDIN9S*, *AtPMMS*, *AtVTC1S*, *AtGMES*, *AtVTC2S*, *AtVTC4S*, *AtGalDHS*, *AtGLDHS*, | PYB539 |
| B5703^*^ | Amp^R^ Kan^R^ | */* | pYB5703 |
| B5702^*^ | Amp^R^ | */* | pYB5702 |

*B5703:used as control strain of B539; B5702: used as control strain of B9240.

**Fig. S1**: UPLC-MS/MS SIM chromatogram analysis of vitamine C from strain B3379 at 8h induction with IPTG and arabinose (upper: M/Z175; middle: M/Z 115; bottom:

M/Z 87).


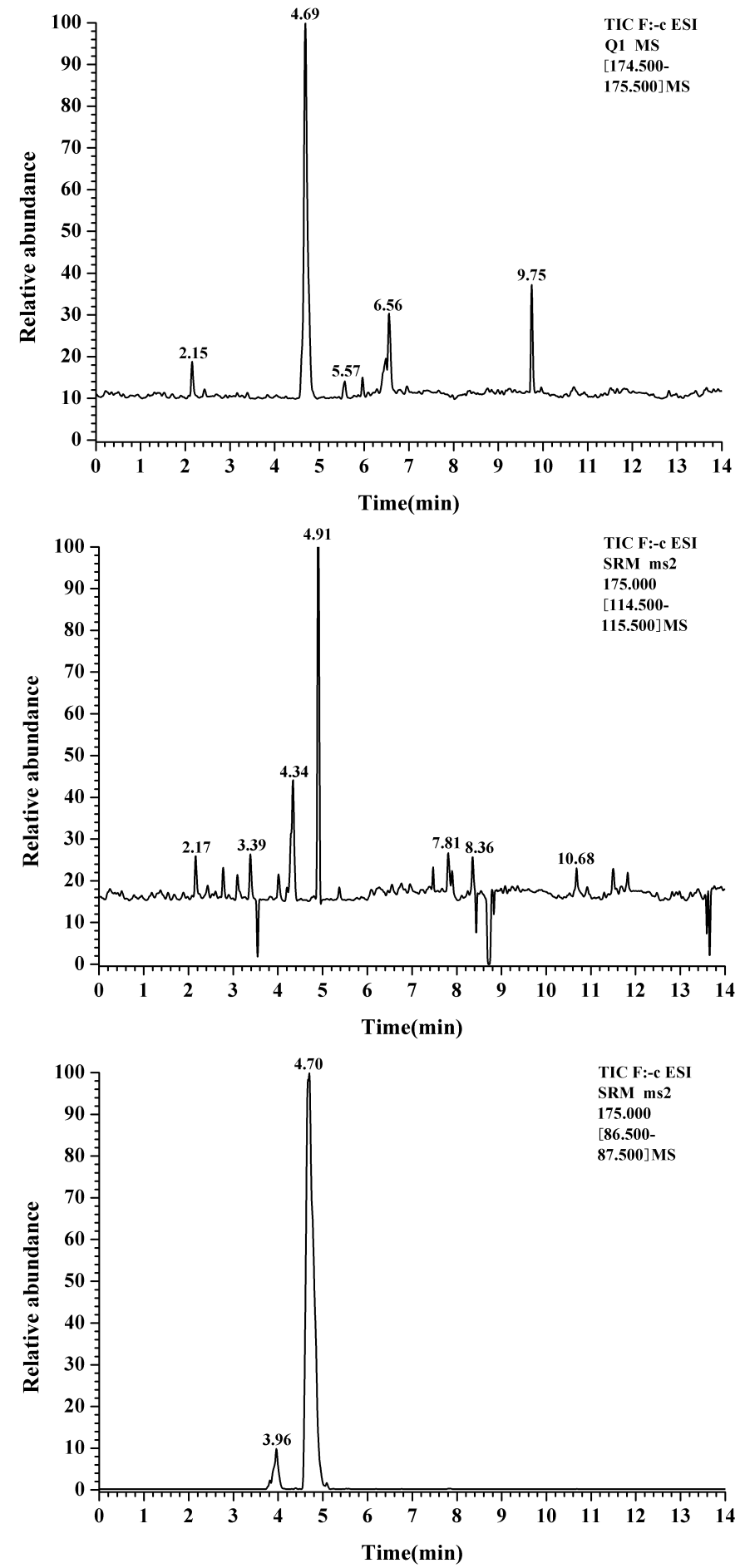


**Fig. S2**: UPLC-MS/MS SIM chromatogram analysis of standard vitamine C (upper: M/Z175; middle: M/Z 115; bottom: M/Z 87).

**
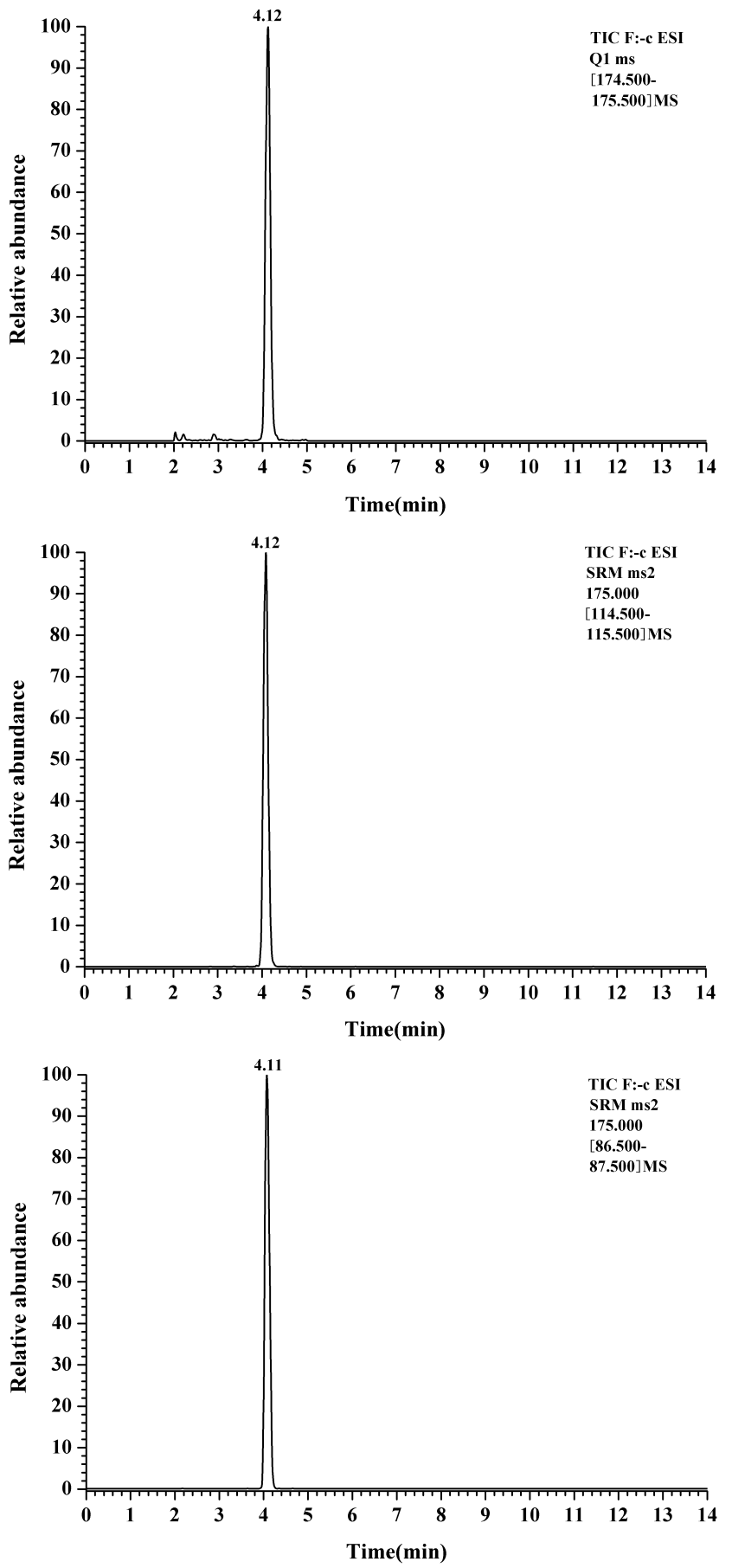
**

**Fig. S3**: UPLC-MS/MS SIM chromatogram analysis of standard U-^13^C-vitamine C (upper: M/Z181; middle: M/Z 119; bottom: M/Z 90).

**
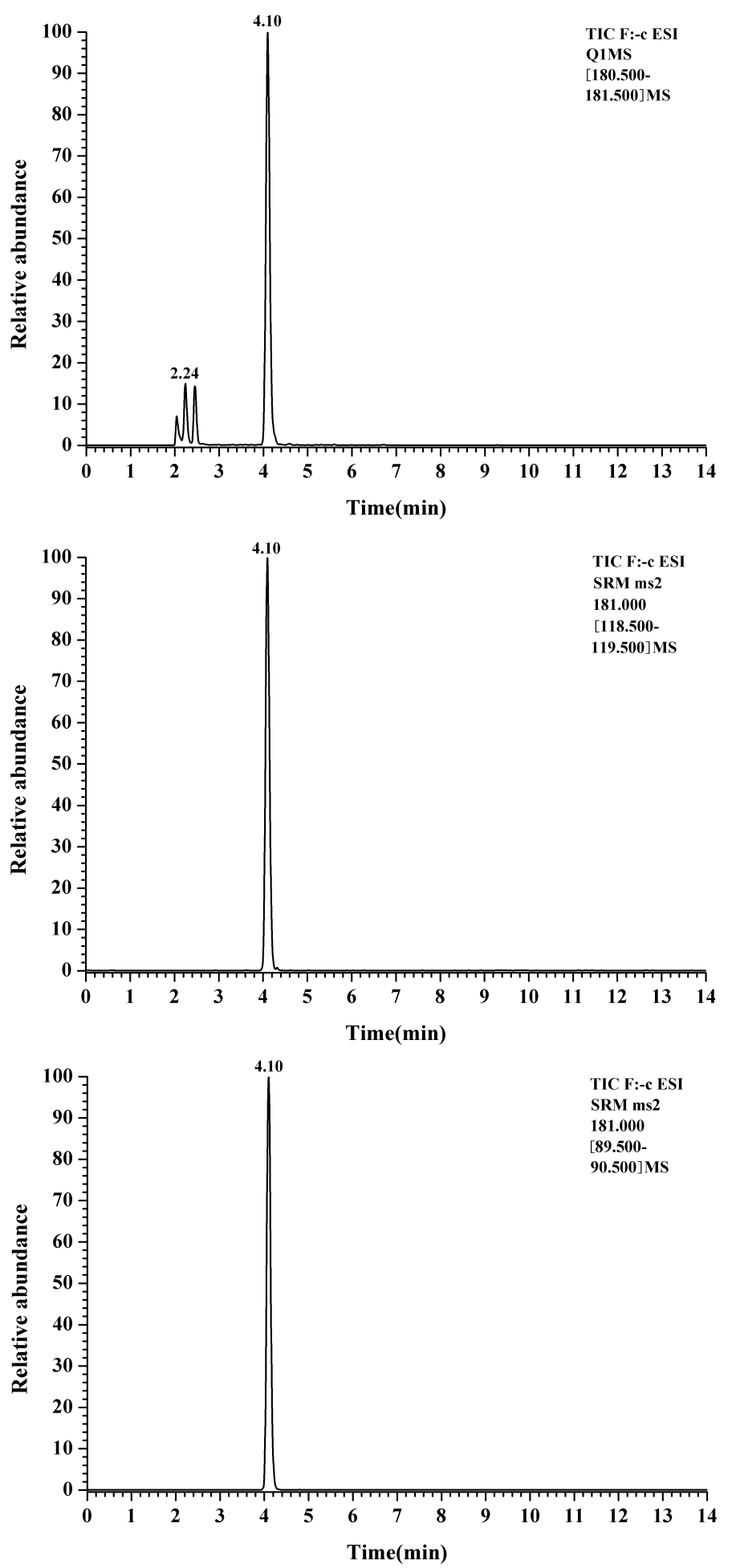
**

**Fig.**S4: Expression of the transgenes in transformant by PCR using DNA as template (1：*AtHXK1S*；2：*AtPGIS*；3：*AtDIN9S*； 4：*AtPMMS*；5：*AtVTC1S*；6：*AtGMES*；7：*AtVTC2S*；8：*AtVTC4S*；9：*AtGalDHS*； 10：*AtGLDHS*. M: marker) .

**
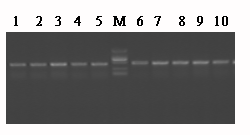
**

**Fig. S5**: The presumably synthesis pathway (green) of L-galactono-1,4-lactone in strain B5703 (KEGG map00053: Ascorbate and aldarate metabolis).

**
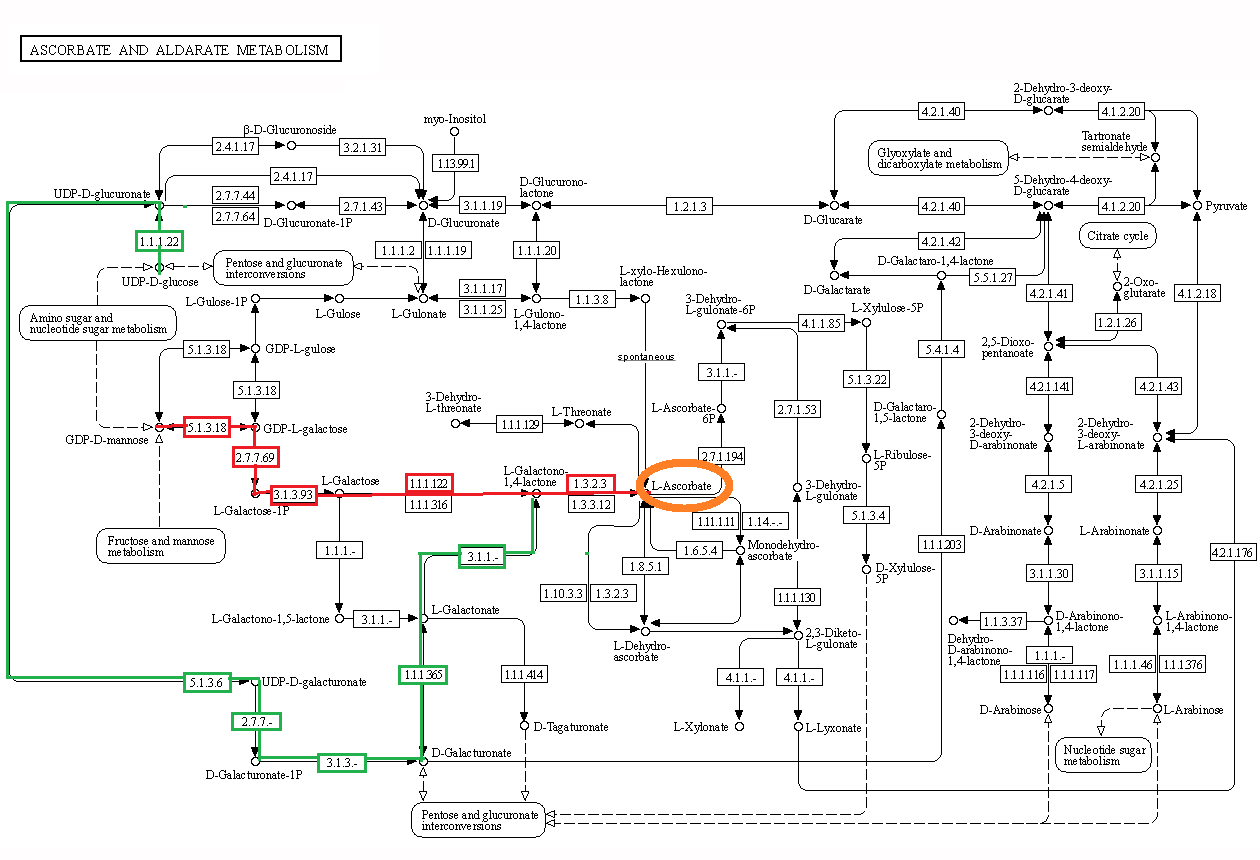
**

**Notes 1**: The sequence information after codon optimization and the primers used for gene synthesize through PCR-based two-step DNA synthesis (PTDS) strategies as follow:

***AtHXK1IS:***

ATGGGTAAGGTTGCTGTTGGAGCAACTGTTGTTTGCACTGCTGCTGTTTGTGCAGTTGCTGTTCTCGTTGTTAGAAGAAGAATGCAGTCTTCTGGTAAGTGGGGTAGAGTTCTCGCTATCCTCAAGGCATTCGAAGAGGACTGCGCAACTCCAATCTCCAAGCTCAGACAAGTTGCTGACGCTATGACTGTCGAGATGCACGCTGGACTCGCATCTGATGGTGGTTCCAAGCTCAAGATGCTCATCTCCTACGTTGACAACCTCCCATCTGGTGACGAGAAGGGACTCTTCTACGCACTCGACCTCGGTGGAACCAACTTCAGAGTCATGAGAGTCCTCCTCGGAGGAAAGCAGGAGAGAGTTGTTAAGCAGGAGTTCGAGGAGGTTTCCATCCCACCACACTTGATGACTGGTGGATCTGACGAACTCTTCAACTTCATCGCTGAAGCACTCGCAAAGTTCGTTGCTACCGAGTGTGAAGACTTCCACCTCCCAGAAGGCAGACAGAGAGAGTTGGGTTTCACCTTCTCCTTCCCTGTTAAGCAGACCTCTCTCTCCTCTGGATCTCTCATCAAGTGGACCAAGGGATTCTCTATCGAGGAGGCAGTCGGACAGGACGTTGTTGGAGCACTCAACAAGGCTCTTGAGAGAGTTGGTCTCGACATGAGAATCGCAGCACTCGTCAACGACACTGTTGGAACCCTCGCAGGTGGTAGATACTACAACCCTGACGTTGTTGCTGCTGTCATCTTGGGTACTGGAACCAACGCAGCATACGTCGAGAGAGCAACCGCAATCCCAAAGTGGCACGGTCTCCTCCCAAAGTCCGGAGAGATGGTTATCAACATGGAGTGGGGAAACTTCAGATCCTCCCACCTCCCATTGACTGAGTTCGACCACACCCTCGACTTCGAATCTCTCAACCCAGGTGAGCAGATCCTTGAGAAGATCATCTCTGGCATGTACTTGGGAGAGATCCTAAGAAGAGTTCTCCTCAAGATGGCTGAGGATGCTGCTTTCTTCGGAGACACTGTTCCATCCAAGCTCAGAATCCCATTCATCATCAGAACTCCACACATGTCTGCTATGCACAACGACACCTCTCCAGACTTGAAGATCGTTGGTTCCAAGATCAAGGACATCTTGGAGGTTCCAACCACCTCTCTCAAGATGAGAAAAGTCGTCATCTCTCTCTGCAACATCATCGCAACCAGAGGAGCTAGACTCTCTGCTGCTGGAATCTACGGAATCCTCAAGAAGCTCGGAAGAGACACCACCAAGGACGAGGAGGTTCAGAAGTCTGTCATCGCTATGGATGGTGGACTCTTCGAGCACTACACCCAGTTCTCTGAGTGCATGGAGTCTTCCCTCAAGGAGTTGCTCGGAGACGAGGCTTCTGGATCTGTTGAGGTTACTCACTCCAACGACGGATCTGGTATCGGAGCAGCACTCCTCGCTGCTTCCCACTCCCTCTACCTCGAAGACTCCTAA

1. ATHXK1S-1: Tm=54, 60mer

ATG,GGT,AAG,GTT,GCT,GTT,GGA,GCA,ACT,GTT,GTT,TGC,ACT,GCT,GCT,GTT,TGT,GCA,GTT,GCT

2. ATHXK1S-2: Tm=54, 60mer

ACT,TAC,CAG,AAG,ACT,GCA,TTC,TTC,TTC,TAA,CAA,CGA,GAA,CAG,CAA,CTG,CAC,AAA,CAG,CAG

3. ATHXK1S-3: Tm=54, 60mer

AAT,GCA,GTC,TTC,TGG,TAA,GTG,GGG,TAG,AGT,TCT,CGC,TAT,CCT,CAA,GGC,ATT,CGA,AGA,GGA

4. ATHXK1S-4: Tm=54, 60mer

GTC,AGC,AAC,TTG,TCT,GAG,CTT,GGA,GAT,TGG,AGT,TGC,GCA,GTC,CTC,TTC,GAA,TGC,CTT,GAG

5. ATHXK1S-5: Tm=54, 60mer

AGC,TCA,GAC,AAG,TTG,CTG,ACG,CTA,TGA,CTG,TCG,AGA,TGC,ACG,CTG,GAC,TCG,CAT,CTG,ATG

6. ATHXK1S-6: Tm=54, 60mer

TTG,TCA,ACG,TAG,GAG,ATG,AGC,ATC,TTG,AGC,TTG,GAA,CCA,CCA,TCA,GAT,GCG,AGT,CCA,GCG

7. ATHXK1S-7: Tm=54, 60mer

CTC,ATC,TCC,TAC,GTT,GAC,AAC,CTC,CCA,TCT,GGT,GAC,GAG,AAG,GGA,CTC,TTC,TAC,GCA,CTC

8. ATHXK1S-8: Tm=54, 60mer

GGA,GGA,CTC,TCA,TGA,CTC,TGA,AGT,TGG,TTC,CAC,CGA,GGT,CGA,GTG,CGT,AGA,AGA,GTC,CCT

9. ATHXK1S-9: Tm=54, 60mer

CAG,AGT,CAT,GAG,AGT,CCT,CCT,CGG,AGG,AAA,GCA,GGA,GAG,AGT,TGT,TAA,GCA,GGA,GTT,CGA

10. ATHXK1S-10: Tm=54, 60mer

AGA,TCC,ACC,AGT,CAT,CAA,GTG,TGG,TGG,GAT,GGA,AAC,CTC,CTC,GAA,CTC,CTG,CTT,AAC,AAC

11. ATHXK1S-11: Tm=54, 60mer

ACT,TGA,TGA,CTG,GTG,GAT,CTG,ACG,AAC,TCT,TCA,ACT,TCA,TCG,CTG,AAG,CAC,TCG,CAA,AGT

12. ATHXK1S-12: Tm=54, 60mer

CCT,TCT,GGG,AGG,TGG,AAG,TCT,TCA,CAC,TCG,GTA,GCA,ACG,AAC,TTT,GCG,AGT,GCT,TCA,GCG

13. ATHXK1S-13: Tm=54, 60mer

GAC,TTC,CAC,CTC,CCA,GAA,GGC,AGA,CAG,AGA,GAG,TTG,GGT,TTC,ACC,TTC,TCC,TTC,CCT,GTT

14. ATHXK1S-14: Tm=54, 60mer

TCC,ACT,TGA,TGA,GAG,ATC,CAG,AGG,AGA,GAG,AGG,TCT,GCT,TAA,CAG,GGA,AGG,AGA,AGG,TGA

15. ATHXK1S-15: Tm=54, 60mer

TGG,ATC,TCT,CAT,CAA,GTG,GAC,CAA,GGG,ATT,CTC,TAT,CGA,GGA,GGC,AGT,CGG,ACA,GGA,CGT

16. ATHXK1S-16: Tm=54, 60mer

GAG,ACC,AAC,TCT,CTC,AAG,AGC,CTT,GTT,GAG,TGC,TCC,AAC,AAC,GTC,CTG,TCC,GAC,TGC,CTC

17. ATHXK1S-17: Tm=54, 60mer

CTC,TTG,AGA,GAG,TTG,GTC,TCG,ACA,TGA,GAA,TCG,CAG,CAC,TCG,TCA,ACG,ACA,CTG,TTG,GAA

18. ATHXK1S-18: Tm=54, 60mer

GCA,ACA,ACG,TCA,GGG,TTG,TAG,TAT,CTA,CCA,CCT,GCG,AGG,GTT,CCA,ACA,GTG,TCG,TTG,ACG

19. ATHXK1S-19: Tm=54, 60mer

TAC,AAC,CCT,GAC,GTT,GTT,GCT,GCT,GTC,ATC,TTG,GGT,ACT,GGA,ACC,AAC,GCA,GCA,TAC,GTC

20. ATHXK1S-20: Tm=54, 60mer

GGA,GGA,GAC,CGT,GCC,ACT,TTG,GGA,TTG,CGG,TTG,CTC,TCT,CGA,CGT,ATG,CTG,CGT,TGG,TTC

21. ATHXK1S-21: Tm=54, 60mer

AAA,GTG,GCA,CGG,TCT,CCT,CCC,AAA,GTC,CGG,AGA,GAT,GGT,TAT,CAA,CAT,GGA,GTG,GGG,AAA

22. ATHXK1S-22: Tm=54, 60mer

GTG,GTC,GAA,CTC,AGT,CAA,TGG,GAG,GTG,GGA,GGA,TCT,GAA,GTT,TCC,CCA,CTC,CAT,GTT,GAT

23. ATHXK1S-23: Tm=54, 60mer

CAT,TGA,CTG,AGT,TCG,ACC,ACA,CCC,TCG,ACT,TCG,AAT,CTC,TCA,ACC,CAG,GTG,AGC,AGA,TCC

24. ATHXK1S-24: Tm=54, 60mer

AGG,ATC,TCT,CCC,AAG,TAC,ATG,CCA,GAG,ATG,ATC,TTC,TCA,AGG,ATC,TGC,TCA,CCT,GGG,TTG

25. ATHXK1S-25: Tm=54, 60mer

ATG,TAC,TTG,GGA,GAG,ATC,CTA,AGA,AGA,GTT,CTC,CTC,AAG,ATG,GCT,GAG,GAT,GCT,GCT,TTC

26. ATHXK1S-26: Tm=54, 60mer

TGA,ATG,GGA,TTC,TGA,GCT,TGG,ATG,GAA,CAG,TGT,CTC,CGA,AGA,AAG,CAG,CAT,CCT,CAG,CCA

27. ATHXK1S-27: Tm=54, 60mer

CAA,GCT,CAG,AAT,CCC,ATT,CAT,CAT,CAG,AAC,TCC,ACA,CAT,GTC,TGC,TAT,GCA,CAA,CGA,CAC

28. ATHXK1S-28: Tm=54, 60mer

GTC,CTT,GAT,CTT,GGA,ACC,AAC,GAT,CTT,CAA,GTC,TGG,AGA,GGT,GTC,GTT,GTG,CAT,AGC,AGA

29. ATHXK1S-29: Tm=54, 60mer

TTG,GTT,CCA,AGA,TCA,AGG,ACA,TCT,TGG,AGG,TTC,CAA,CCA,CCT,CTC,TCA,AGA,TGA,GAA,AAG

30. ATHXK1S-30: Tm=54, 60mer

GCT,CCT,CTG,GTT,GCG,ATG,ATG,TTG,CAG,AGA,GAG,ATG,ACG,ACT,TTT,CTC,ATC,TTG,AGA,GAG

31. ATHXK1S-31: Tm=54, 60mer

ATC,ATC,GCA,ACC,AGA,GGA,GCT,AGA,CTC,TCT,GCT,GCT,GGA,ATC,TAC,GGA,ATC,CTC,AAG,AAG

32. ATHXK1S-32: Tm=54, 60mer

ACT,TCT,GAA,CCT,CCT,CGT,CCT,TGG,TGG,TGT,CTC,TTC,CGA,GCT,TCT,TGA,GGA,TTC,CGT,AGA

33. ATHXK1S-33: Tm=54, 60mer

GGA,CGA,GGA,GGT,TCA,GAA,GTC,TGT,CAT,CGC,TAT,GGA,TGG,TGG,ACT,CTT,CGA,GCA,CTA,CAC

34. ATHXK1S-34: Tm=54, 60mer

CAA,CTC,CTT,GAG,GGA,AGA,CTC,CAT,GCA,CTC,AGA,GAA,CTG,GGT,GTA,GTG,CTC,GAA,GAG,TCC

35. ATHXK1S-35: Tm=54, 60mer

AGT,CTT,CCC,TCA,AGG,AGT,TGC,TCG,GAG,ACG,AGG,CTT,CTG,GAT,CTG,TTG,AGG,TTA,CTC,ACT

36. ATHXK1S-36: Tm=54, 60mer

GCA,GCG,AGG,AGT,GCT,GCT,CCG,ATA,CCA,GAT,CCG,TCG,TTG,GAG,TGA,GTA,ACC,TCA,ACA,GAT

37. ATHXK1S-37: Tm=54, 51mer

TTA,GGA,GTC,TTC,GAG,GTA,GAG,GGA,GTG,GGA,AGC,AGC,GAG,GAG,TGC,TGC,TCC

***ATPGIS:*** ATGGCATCTCTCTCTGGACTCTACTCTTCTTCTCCATCTCTCAAGCCTGCTAAGAACCACTCCTTCAAGGCATTGCCTGCACAGTCCAGAGACTCTTTCTCTTTCCCACACACCTCTAAGCCAACCAACCTCCCATTGACCCTCTCTTCTGCTAGATCCGTCGCAAGAGACATCTCTCATGCTGACTCTAAGAAGGAGCTTCTCAAGGACCCTGACGCATTGTGGAAGAGATACCTCGACTGGTTCTACCAGCAGAAGGAGCTTGGATTGTACCTCGACATCTCCAGAGTCGGATTTACCGATGAGTTTGTGGCTGAAATGGAGCCAAGGTTTCAAGCTGCGTTTAAGGCTATGGAGGATCTTGAAAAAGGGTCTATAGCGAATCCTGATGAAGGAAGAATGGTTGGACATTACTGGCTTAGGAACTCTAAGCTCGCACCTAAGCCTACTTTAAAGACCTTGATCGAGAACACACTTGATTCTATTTGCGCTTTCTCCGACGACATAATCTCTGGAAAGATAAAGCCACCATCTTCTCCTGAGGGTCGTTTTACTCAGATACTTTCTGTTGGCATTGGAGGCTCGGCTCTTGGACCTCAATTTGTCGCTGAGGCACTCGCTCCTGATAATCCTCCATTGAAGATAAGATTCATTGACAACACCGACCCTGCTGGAATTGATCATCAGATTGCACAACTTGGGCCAGAGCTGGCCTCGACTTTGGTTGTTGTCATCTCTAAGTCTGGAGGAACCCCTGAGACCAGAAACGGACTCCTCGAAGTCCAGAAGGCATTCAGAGAGGCTGGTCTCAACTTCGCAAAGCAGGGTGTTGCAATCACTCAAGAGAACTCCTTGCTCGACAACACTGCAAGAATCGAGGGATGGCTTGCTAGATTCCCTATGTACGACTGGGTTGGTGGAAGAACCTCCATCATGTCTGCTGTTGGTCTCCTTCCAGCAGCACTTCAGGGAATCAACGTTAGAGAGATGCTCACTGGTGCTGCTTTGATGGATGAGGCTACCAGAACCACCTCTATCAAGAACAACCCTGCTGCACTCTTGGCTATGTGCTGGTACTGGGCTTCCAACGGTGTCGGTTCCAAGGACATGGTTGTCCTCCCATACAAGGACTCTCTCTTGCTCTTCTCCAGATACCTCCAGCAGCTCGTCATGGAGTCTCTTGGAAAGGAGTTCGACCTCGACGGTAACACTGTCAACCAGGGATTGACTGTCTACGGAAACAAGGGTTCCACTGACCAACACGCATACATCCAGCAGCTCAGAGACGGAGTTCACAACTTCTTCGCAACCTTCATCGAGGTTCTCAGAGACAGACCACCTGGTCACGACTGGGAGCTTGAGCCAGGTGTCACCTGTGGAGACTACCTCTTCGGAATGCTCCAGGGAACCAGATCCGCTCTCTACGCAAACGGTAGAGAGTCCATCTCTGTTACCATCCAGGAAGTCACTCCAACCTCTGTTGGTGCTATCATCGCTCTCTACGAGAGAGCTGTTGGTTTGTACGCATCTATCGTCAACATCAACGCTTACCACCAGCCTGGTGTTGAGGCTGGTAAGAAGGCAGCAGCAGAGGTTCTCGCACTCCAGAAGAGAGTTCTCTCTGTTCTCAACGAGGCAACCTGCAAGGACCCTGTTGAGCCACTCACCCTCGAAGAGATCGCTGACAGATGTCACGCTCCTGAGGAGATTGAGATGATCTACAAGATCATTGCACACATGTCTGCAAACGACAGAGTTCTCATCGCAGAAGGAAACTGTGGATCTCCAAGATCCATCAAGGTCTACCTCGGTGAGTGCAACGTCGATGACCTCTACGCATAA

1. ATPGI1S-1: Tm=54, 60mer

ATG,GCA,TCT,CTC,TCT,GGA,CTC,TAC,TCT,TCT,TCT,CCA,TCT,CTC,AAG,CCT,GCT,AAG,AAC,CAC

2. ATPGI1S-2: Tm=54, 60mer

AGA,AAG,AGT,CTC,TGG,ACT,GTG,CAG,GCA,ATG,CCT,TGA,AGG,AGT,GGT,TCT,TAG,CAG,GCT,TGA

3. ATPGI1S-3: Tm=54, 60mer

ACA,GTC,CAG,AGA,CTC,TTT,CTC,TTT,CCC,ACA,CAC,CTC,TAA,GCC,AAC,CAA,CCT,CCC,ATT,GAC

4. ATPGI1S-4: Tm=54, 60mer

ATG,AGA,GAT,GTC,TCT,TGC,GAC,GGA,TCT,AGC,AGA,AGA,GAG,GGT,CAA,TGG,GAG,GTT,GGT,TGG

5. ATPGI1S-5: Tm=54, 60mer

TCG,CAA,GAG,ACA,TCT,CTC,ATG,CTG,ACT,CTA,AGA,AGG,AGC,TTC,TCA,AGG,ACC,CTG,ACG,CAT

6. ATPGI1S-6: Tm=54, 60mer

TCC,TTC,TGC,TGG,TAG,AAC,CAG,TCG,AGG,TAT,CTC,TTC,CAC,AAT,GCG,TCA,GGG,TCC,TTG,AGA

7. ATPGI1S-7: Tm=54, 60mer

TGG,TTC,TAC,CAG,CAG,AAG,GAG,CTT,GGA,TTG,TAC,CTC,GAC,ATC,TCC,AGA,GTC,GGA,TTT,ACC

8. ATPGI1S-8: Tm=54, 60mer

CAG,CTT,GAA,ACC,TTG,GCT,CCA,TTT,CAG,CCA,CAA,ACT,CAT,CGG,TAA,ATC,CGA,CTC,TGG,AGA

9. ATPGI1S-9: Tm=54, 60mer

GGA,GCC,AAG,GTT,TCA,AGC,TGC,GTT,TAA,GGC,TAT,GGA,GGA,TCT,TGA,AAA,AGG,GTC,TAT,AGC

10. ATPGI1S-10: Tm=54, 60mer

AAG,CCA,GTA,ATG,TCC,AAC,CAT,TCT,TCC,TTC,ATC,AGG,ATT,CGC,TAT,AGA,CCC,TTT,TTC,AAG

11. ATPGI1S-11: Tm=54, 60mer

TGG,TTG,GAC,ATT,ACT,GGC,TTA,GGA,ACT,CTA,AGC,TCG,CAC,CTA,AGC,CTA,CTT,TAA,AGA,CCT

12. ATPGI1S-12: Tm=54, 60mer

TCG,GAG,AAA,GCG,CAA,ATA,GAA,TCA,AGT,GTG,TTC,TCG,ATC,AAG,GTC,TTT,AAA,GTA,GGC,TTA

13. ATPGI1S-13: Tm=54, 60mer

TCT,ATT,TGC,GCT,TTC,TCC,GAC,GAC,ATA,ATC,TCT,GGA,AAG,ATA,AAG,CCA,CCA,TCT,TCT,CCT

14. ATPGI1S-14: Tm=54, 60mer

CTC,CAA,TGC,CAA,CAG,AAA,GTA,TCT,GAG,TAA,AAC,GAC,CCT,CAG,GAG,AAG,ATG,GTG,GCT,TTA

15. ATPGI1S-15: Tm=54, 60mer

ACT,TTC,TGT,TGG,CAT,TGG,AGG,CTC,GGC,TCT,TGG,ACC,TCA,ATT,TGT,CGC,TGA,GGC,ACT,CGC

16. ATPGI1S-16: Tm=54, 60mer

GTT,GTC,AAT,GAA,TCT,TAT,CTT,CAA,TGG,AGG,ATT,ATC,AGG,AGC,GAG,TGC,CTC,AGC,GAC,AAA

17. ATPGI1S-17: Tm=54, 60mer

AGA,TAA,GAT,TCA,TTG,ACA,ACA,CCG,ACC,CTG,CTG,GAA,TTG,ATC,ATC,AGA,TTG,CAC,AAC,TTG

18. ATPGI1S-18: Tm=54, 60mer

TTA,GAG,ATG,ACA,ACA,ACC,AAA,GTC,GAG,GCC,AGC,TCT,GGC,CCA,AGT,TGT,GCA,ATC,TGA,TGA

19. ATPGI1S-19: Tm=54, 60mer

TTG,GTT,GTT,GTC,ATC,TCT,AAG,TCT,GGA,GGA,ACC,CCT,GAG,ACC,AGA,AAC,GGA,CTC,CTC,GAA

20. ATPGI1S-20: Tm=54, 60mer

TTG,CGA,AGT,TGA,GAC,CAG,CCT,CTC,TGA,ATG,CCT,TCT,GGA,CTT,CGA,GGA,GTC,CGT,TTC,TGG

21. ATPGI1S-21: Tm=54, 60mer

GGC,TGG,TCT,CAA,CTT,CGC,AAA,GCA,GGG,TGT,TGC,AAT,CAC,TCA,AGA,GAA,CTC,CTT,GCT,CGA

22. ATPGI1S-22: Tm=54, 60mer

AGG,GAA,TCT,AGC,AAG,CCA,TCC,CTC,GAT,TCT,TGC,AGT,GTT,GTC,GAG,CAA,GGA,GTT,CTC,TTG

23. ATPGI1S-23: Tm=54, 60mer

GAT,GGC,TTG,CTA,GAT,TCC,CTA,TGT,ACG,ACT,GGG,TTG,GTG,GAA,GAA,CCT,CCA,TCA,TGT,CTG

24. ATPGI1S-24: Tm=54, 60mer

ACG,TTG,ATT,CCC,TGA,AGT,GCT,GCT,GGA,AGG,AGA,CCA,ACA,GCA,GAC,ATG,ATG,GAG,GTT,CTT

25. ATPGI1S-25: Tm=54, 60mer

GCA,CTT,CAG,GGA,ATC,AAC,GTT,AGA,GAG,ATG,CTC,ACT,GGT,GCT,GCT,TTG,ATG,GAT,GAG,GCT

26. ATPGI1S-26: Tm=54, 60mer

AGA,GTG,CAG,CAG,GGT,TGT,TCT,TGA,TAG,AGG,TGG,TTC,TGG,TAG,CCT,CAT,CCA,TCA,AAG,CAG

27. ATPGI1S-27: Tm=54, 60mer

GAA,CAA,CCC,TGC,TGC,ACT,CTT,GGC,TAT,GTG,CTG,GTA,CTG,GGC,TTC,CAA,CGG,TGT,CGG,TTC

28. ATPGI1S-28: Tm=54, 60mer

CAA,GAG,AGA,GTC,CTT,GTA,TGG,GAG,GAC,AAC,CAT,GTC,CTT,GGA,ACC,GAC,ACC,GTT,GGA,AGC

29. ATPGI1S-29: Tm=54, 60mer

CAT,ACA,AGG,ACT,CTC,TCT,TGC,TCT,TCT,CCA,GAT,ACC,TCC,AGC,AGC,TCG,TCA,TGG,AGT,CTC

30. ATPGI1S-30: Tm=54, 60mer

TGG,TTG,ACA,GTG,TTA,CCG,TCG,AGG,TCG,AAC,TCC,TTT,CCA,AGA,GAC,TCC,ATG,ACG,AGC,TGC

31. ATPGI1S-31: Tm=54, 60mer

GAC,GGT,AAC,ACT,GTC,AAC,CAG,GGA,TTG,ACT,GTC,TAC,GGA,AAC,AAG,GGT,TCC,ACT,GAC,CAA

32. ATPGI1S-32: Tm=54, 60mer

AGT,TGT,GAA,CTC,CGT,CTC,TGA,GCT,GCT,GGA,TGT,ATG,CGT,GTT,GGT,CAG,TGG,AAC,CCT,TGT

33. ATPGI1S-33: Tm=54, 60mer

CAG,AGA,CGG,AGT,TCA,CAA,CTT,CTT,CGC,AAC,CTT,CAT,CGA,GGT,TCT,CAG,AGA,CAG,ACC,ACC

34. ATPGI1S-34: Tm=54, 60mer

TCC,ACA,GGT,GAC,ACC,TGG,CTC,AAG,CTC,CCA,GTC,GTG,ACC,AGG,TGG,TCT,GTC,TCT,GAG,AAC

35. ATPGI1S-35: Tm=54, 60mer

AGC,CAG,GTG,TCA,CCT,GTG,GAG,ACT,ACC,TCT,TCG,GAA,TGC,TCC,AGG,GAA,CCA,GAT,CCG,CTC

36. ATPGI1S-36: Tm=54, 60mer

TGG,ATG,GTA,ACA,GAG,ATG,GAC,TCT,CTA,CCG,TTT,GCG,TAG,AGA,GCG,GAT,CTG,GTT,CCC,TGG

37. ATPGI1S-37: Tm=54, 60mer

TCC,ATC,TCT,GTT,ACC,ATC,CAG,GAA,GTC,ACT,CCA,ACC,TCT,GTT,GGT,GCT,ATC,ATC,GCT,CTC

38. ATPGI1S-38: Tm=54, 60mer

TGT,TGA,CGA,TAG,ATG,CGT,ACA,AAC,CAA,CAG,CTC,TCT,CGT,AGA,GAG,CGA,TGA,TAG,CAC,CAA

39. ATPGI1S-39: Tm=54, 60mer

GTA,CGC,ATC,TAT,CGT,CAA,CAT,CAA,CGC,TTA,CCA,CCA,GCC,TGG,TGT,TGA,GGC,TGG,TAA,GAA

40. ATPGI1S-40: Tm=54, 60mer

GAG,AAC,TCT,CTT,CTG,GAG,TGC,GAG,AAC,CTC,TGC,TGC,TGC,CTT,CTT,ACC,AGC,CTC,AAC,ACC

41. ATPGI1S-41: Tm=54, 60mer

CAC,TCC,AGA,AGA,GAG,TTC,TCT,CTG,TTC,TCA,ACG,AGG,CAA,CCT,GCA,AGG,ACC,CTG,TTG,AGC

42. ATPGI1S-42: Tm=54, 60mer

GGA,GCG,TGA,CAT,CTG,TCA,GCG,ATC,TCT,TCG,AGG,GTG,AGT,GGC,TCA,ACA,GGG,TCC,TTG,CAG

43. ATPGI1S-43: Tm=54, 60mer

GCT,GAC,AGA,TGT,CAC,GCT,CCT,GAG,GAG,ATT,GAG,ATG,ATC,TAC,AAG,ATC,ATT,GCA,CAC,ATG

44. ATPGI1S-44: Tm=54, 60mer

CAC,AGT,TTC,CTT,CTG,CGA,TGA,GAA,CTC,TGT,CGT,TTG,CAG,ACA,TGT,GTG,CAA,TGA,TCT,TGT

45. ATPGI1S-45: Tm=54, 60mer

CAT,CGC,AGA,AGG,AAA,CTG,TGG,ATC,TCC,AAG,ATC,CAT,CAA,GGT,CTA,CCT,CGG,TGA,GTG,CAA

46. ATPGI1S-46: Tm=54, 42mer

TTA,TGC,GTA,GAG,GTC,ATC,GAC,GTT,GCA,CTC,ACC,GAG,GTA,GAC

***AtDIN9S:***  ATGGGAGCAGACGCAATCCAGACTAACGGTCACGACCAAGCTAAGTTGACTGGAGGAGAAGAGATCCAGAGACTCAGATGCTTCGTCAAGAACTACGAGTGGGGTAAGCTCGGACCTGAGTCTCTCGTTGCTAGACTCCAGGAGGCTAACACTGGACAGAGAGTTGACTCTGAGATCCCATACGCTGAGTTCTGGATGGGTACTCACGAGTCTGGACCATCTCACGTCGAGTTCGGATCTGGTCACGGTGTCTCTGACAAGTGCATGGTCACTCTCAAGTCTTGGGTCTTGGACAACCCTAACTTGCTCGGTTCCAAGGTTGTTGACAAGTGGGGATGTGACCTCCCATTCCTCTTCAAGGTTCTCTCTGTCACCAAGGCATTGTCTATCCAAGCACACCCAAACAAGGCATTGGCAGAGAAGTTGCACAGAGAGGACCCACTCCTCTACAGAGACAACAACCACAAGCCTGAGATCGCTCTCGCTGTCACTCCATTCCAAGCACTCTGCGGATTCGTCACTCTCAAGGAACTCAAGGAGGTCATCACCAACGTTCCAGAGATCACCGAGCTTGTCGGTTCCAAGGCTGCTGACCAAATCTTCAACGTTCACGAACACGACGAAGACGAGAGAATCAAGTCCGTCGTCAGACTCATCTTCACCCAGTTGATGTCTGCATCCAACAACGAGACCAAGCAGGTTGTCTCCAGAATGAAGAACAGACTCCTCTTGGAGACCAAGCACAGAGAACTCTCCGAGAAGGAGAAGTTGGTCTTGGAGCTTGAGAAGCAGTACACTGGTGACATCGGTGTCATCTCTGCATTCTTCTTCAACTACGTCAAGCTCAACCCTGGAGAGGCTCTCTACTTGGACGCTAACGAGCCACACGCATACATCTCTGGTGACTGTGTCGAGTGCATGGCTGCTTCTGACAACGTCGTCAGAGCTGGTCTCACTCCTAAGCACAGAGACGTCCAGACCCTCTGCTCCATGCTCACCTACAAGTTGGGATACCCTGAGATCCTCAAGGGATTCCCTCTCACTCCATACGTCACCAGATACCTCCCTCCATTCGACGAGTTCGAGGTTGACCACTGTGACCTCCCTAGAGGAAAGTCCACCGTCTTCCCAGCAGTTCCAGGACCTTCTGTCTACTTGGTCATCGAAGGAAAGGGACAGTTGAGAACCGGATCTTCCAAGGTCTTGGTCAACAGAGGAGACGTCTTGTTCGTTCCTGCTGACATCGAGATCCACGTCACTGGAGAGTCTGATGTCATGAAGCTCTACAGAGCTGGAGTCTCTTCCAGATTCTTCCAGACCTTGTAA

1. ATDIN9S-1: Tm=54, 60mer

ATG,GGA,GCA,GAC,GCA,ATC,CAG,ACT,AAC,GGT,CAC,GAC,CAA,GCT,AAG,TTG,ACT,GGA,GGA,GAA

2. ATDIN9S-2: Tm=54, 60mer

ACT,CGT,AGT,TCT,TGA,CGA,AGC,ATC,TGA,GTC,TCT,GGA,TCT,CTT,CTC,CTC,CAG,TCA,ACT,TAG

3. ATDIN9S-3: Tm=54, 60mer

CTT,CGT,CAA,GAA,CTA,CGA,GTG,GGG,TAA,GCT,CGG,ACC,TGA,GTC,TCT,CGT,TGC,TAG,ACT,CCA

4. ATDIN9S-4: Tm=54, 60mer

TGG,GAT,CTC,AGA,GTC,AAC,TCT,CTG,TCC,AGT,GTT,AGC,CTC,CTG,GAG,TCT,AGC,AAC,GAG,AGA

5. ATDIN9S-5: Tm=54, 60mer

GAG,TTG,ACT,CTG,AGA,TCC,CAT,ACG,CTG,AGT,TCT,GGA,TGG,GTA,CTC,ACG,AGT,CTG,GAC,CAT

6. ATDIN9S-6: Tm=54, 60mer

TTG,TCA,GAG,ACA,CCG,TGA,CCA,GAT,CCG,AAC,TCG,ACG,TGA,GAT,GGT,CCA,GAC,TCG,TGA,GTA

7. ATDIN9S-7: Tm=54, 60mer

GGT,CAC,GGT,GTC,TCT,GAC,AAG,TGC,ATG,GTC,ACT,CTC,AAG,TCT,TGG,GTC,TTG,GAC,AAC,CCT

8. ATDIN9S-8: Tm=54, 60mer

CAC,ATC,CCC,ACT,TGT,CAA,CAA,CCT,TGG,AAC,CGA,GCA,AGT,TAG,GGT,TGT,CCA,AGA,CCC,AAG

9. ATDIN9S-9: Tm=54, 60mer

TGT,TGA,CAA,GTG,GGG,ATG,TGA,CCT,CCC,ATT,CCT,CTT,CAA,GGT,TCT,CTC,TGT,CAC,CAA,GGC

10. ATDIN9S-10: Tm=54, 60mer

CTC,TGC,CAA,TGC,CTT,GTT,TGG,GTG,TGC,TTG,GAT,AGA,CAA,TGC,CTT,GGT,GAC,AGA,GAG,AAC

11. ATDIN9S-11: Tm=54, 60mer

CAA,ACA,AGG,CAT,TGG,CAG,AGA,AGT,TGC,ACA,GAG,AGG,ACC,CAC,TCC,TCT,ACA,GAG,ACA,ACA

12. ATDIN9S-12: Tm=54, 60mer

TGG,AAT,GGA,GTG,ACA,GCG,AGA,GCG,ATC,TCA,GGC,TTG,TGG,TTG,TTG,TCT,CTG,TAG,AGG,AGT

13. ATDIN9S-13: Tm=54, 60mer

CTC,GCT,GTC,ACT,CCA,TTC,CAA,GCA,CTC,TGC,GGA,TTC,GTC,ACT,CTC,AAG,GAA,CTC,AAG,GAG

14. ATDIN9S-14: Tm=54, 60mer

AAC,CGA,CAA,GCT,CGG,TGA,TCT,CTG,GAA,CGT,TGG,TGA,TGA,CCT,CCT,TGA,GTT,CCT,TGA,GAG

15. ATDIN9S-15: Tm=54, 60mer

GAT,CAC,CGA,GCT,TGT,CGG,TTC,CAA,GGC,TGC,TGA,CCA,AAT,CTT,CAA,CGT,TCA,CGA,ACA,CGA

16. ATDIN9S-16: Tm=54, 60mer

GAA,GAT,GAG,TCT,GAC,GAC,GGA,CTT,GAT,TCT,CTC,GTC,TTC,GTC,GTG,TTC,GTG,AAC,GTT,GAA

17. ATDIN9S-17: Tm=54, 60mer

CCG,TCG,TCA,GAC,TCA,TCT,TCA,CCC,AGT,TGA,TGT,CTG,CAT,CCA,ACA,ACG,AGA,CCA,AGC,AGG

18. ATDIN9S-18: Tm=54, 60mer

TTG,GTC,TCC,AAG,AGG,AGT,CTG,TTC,TTC,ATT,CTG,GAG,ACA,ACC,TGC,TTG,GTC,TCG,TTG,TTG

19. ATDIN9S-19: Tm=54, 60mer

AGA,CTC,CTC,TTG,GAG,ACC,AAG,CAC,AGA,GAA,CTC,TCC,GAG,AAG,GAG,AAG,TTG,GTC,TTG,GAG

20. ATDIN9S-20: Tm=54, 60mer

CAG,AGA,TGA,CAC,CGA,TGT,CAC,CAG,TGT,ACT,GCT,TCT,CAA,GCT,CCA,AGA,CCA,ACT,TCT,CCT

21. ATDIN9S-21: Tm=54, 60mer

TGA,CAT,CGG,TGT,CAT,CTC,TGC,ATT,CTT,CTT,CAA,CTA,CGT,CAA,GCT,CAA,CCC,TGG,AGA,GGC

22. ATDIN9S-22: Tm=54, 60mer

AGA,GAT,GTA,TGC,GTG,TGG,CTC,GTT,AGC,GTC,CAA,GTA,GAG,AGC,CTC,TCC,AGG,GTT,GAG,CTT

23. ATDIN9S-23: Tm=54, 60mer

AGC,CAC,ACG,CAT,ACA,TCT,CTG,GTG,ACT,GTG,TCG,AGT,GCA,TGG,CTG,CTT,CTG,ACA,ACG,TCG

24. ATDIN9S-24: Tm=54, 60mer

GTC,TGG,ACG,TCT,CTG,TGC,TTA,GGA,GTG,AGA,CCA,GCT,CTG,ACG,ACG,TTG,TCA,GAA,GCA,GCC

25. ATDIN9S-25: Tm=54, 60mer

AAG,CAC,AGA,GAC,GTC,CAG,ACC,CTC,TGC,TCC,ATG,CTC,ACC,TAC,AAG,TTG,GGA,TAC,CCT,GAG

26. ATDIN9S-26: Tm=54, 60mer

ATC,TGG,TGA,CGT,ATG,GAG,TGA,GAG,GGA,ATC,CCT,TGA,GGA,TCT,CAG,GGT,ATC,CCA,ACT,TGT

27. ATDIN9S-27: Tm=54, 60mer

CAC,TCC,ATA,CGT,CAC,CAG,ATA,CCT,CCC,TCC,ATT,CGA,CGA,GTT,CGA,GGT,TGA,CCA,CTG,TGA

28. ATDIN9S-28: Tm=54, 60mer

TGG,AAC,TGC,TGG,GAA,GAC,GGT,GGA,CTT,TCC,TCT,AGG,GAG,GTC,ACA,GTG,GTC,AAC,CTC,GAA

29. ATDIN9S-29: Tm=54, 60mer

CCG,TCT,TCC,CAG,CAG,TTC,CAG,GAC,CTT,CTG,TCT,ACT,TGG,TCA,TCG,AAG,GAA,AGG,GAC,AGT

30. ATDIN9S-30: Tm=54, 60mer

TCT,CCT,CTG,TTG,ACC,AAG,ACC,TTG,GAA,GAT,CCG,GTT,CTC,AAC,TGT,CCC,TTT,CCT,TCG,ATG

31. ATDIN9S-31: Tm=54, 60mer

GTC,TTG,GTC,AAC,AGA,GGA,GAC,GTC,TTG,TTC,GTT,CCT,GCT,GAC,ATC,GAG,ATC,CAC,GTC,ACT

32. ATDIN9S-32: Tm=54, 60mer

AGA,CTC,CAG,CTC,TGT,AGA,GCT,TCA,TGA,CAT,CAG,ACT,CTC,CAG,TGA,CGT,GGA,TCT,CGA,TGT

33. ATDIN9S-33: Tm=54, 46mer

TTA,CAA,GGT,CTG,GAA,GAA,TCT,GGA,AGA,GAC,TCC,AGC,TCT,GTA,GAG,C

***AtPMMS:***  ATGGCTGCAAAGATCCCTGGAGTTATCGCTCTCTTCGACGTTGACGGTACTCTCACTGCACCAAGAAAGGAGGCTACTCCAGAGCTTCTCGACTTCATCAGAGAGTTGAGAAAGGTTGTTACCATCGGAGTTGTTGGTGGATCTGACCTCTCCAAAATCTCTGAGCAGCTTGGCAAGACCGTTACCAACGACTACGACTACTGCTTCTCTGAGAACGGTCTTGTTGCACACAAGGATGGTAAGTCCATCGGAATCCAGTCTCTCAAGCTCCACCTCGGAGACGACAAGCTCAAGGAGTTGATCAACTTCACCCTCCACTACATCGCAGACCTCGACATCCCAATCAAGAGAGGCACCTTCATCGAGTTCAGAAACGGAATGCTCAACGTTTCTCCTATCGGTAGAAACTGCTCTCAGGAGGAGAGAGACGAGTTCGAGAGATACGACAAGGTTCAGAACATCAGACCAAAGATGGTTGCTGAACTCAGAGAGAGATTCGCACACCTCAACCTCACCTTCTCTATCGGAGGACAAATCTCTTTCGACGTCTTCCCAAAGGGTTGGGACAAGACCTACTGCTTGCAGTACCTTGAGGACTTCTCTGAGATCCACTTCTTCGGTGACAAGACCTACGAGGGTGGAAACGACTACGAAATCTACGAGTCTCCAAAGACCATCGGACACTCTGTTACCTCTCCAGACGACACTGTTGCAAAGTGCAAGGCTCTCTTCATGTCTTAA

1. ATPMMS-1: Tm=54, 60mer

ATG,GCT,GCA,AAG,ATC,CCT,GGA,GTT,ATC,GCT,CTC,TTC,GAC,GTT,GAC,GGT,ACT,CTC,ACT,GCA

2. ATPMMS-2: Tm=54, 60mer

TGA,TGA,AGT,CGA,GAA,GCT,CTG,GAG,TAG,CCT,CCT,TTC,TTG,GTG,CAG,TGA,GAG,TAC,CGT,CAA

3. ATPMMS-3: Tm=54, 60mer

AGA,GCT,TCT,CGA,CTT,CAT,CAG,AGA,GTT,GAG,AAA,GGT,TGT,TAC,CAT,CGG,AGT,TGT,TGG,TGG

4. ATPMMS-4: Tm=54, 60mer

GGT,CTT,GCC,AAG,CTG,CTC,AGA,GAT,TTT,GGA,GAG,GTC,AGA,TCC,ACC,AAC,AAC,TCC,GAT,GGT

5. ATPMMS-5: Tm=54, 60mer

CTG,AGC,AGC,TTG,GCA,AGA,CCG,TTA,CCA,ACG,ACT,ACG,ACT,ACT,GCT,TCT,CTG,AGA,ACG,GTC

6. ATPMMS-6: Tm=54, 60mer

GAC,TGG,ATT,CCG,ATG,GAC,TTA,CCA,TCC,TTG,TGT,GCA,ACA,AGA,CCG,TTC,TCA,GAG,AAG,CAG

7. ATPMMS-7: Tm=54, 60mer

AAG,TCC,ATC,GGA,ATC,CAG,TCT,CTC,AAG,CTC,CAC,CTC,GGA,GAC,GAC,AAG,CTC,AAG,GAG,TTG

8. ATPMMS-8: Tm=54, 60mer

GGA,TGT,CGA,GGT,CTG,CGA,TGT,AGT,GGA,GGG,TGA,AGT,TGA,TCA,ACT,CCT,TGA,GCT,TGT,CGT

9. ATPMMS-9: Tm=54, 60mer

CAT,CGC,AGA,CCT,CGA,CAT,CCC,AAT,CAA,GAG,AGG,CAC,CTT,CAT,CGA,GTT,CAG,AAA,CGG,AAT

10. ATPMMS-10: Tm=54, 60mer

CTC,CTG,AGA,GCA,GTT,TCT,ACC,GAT,AGG,AGA,AAC,GTT,GAG,CAT,TCC,GTT,TCT,GAA,CTC,GAT

11. ATPMMS-11: Tm=54, 60mer

GTA,GAA,ACT,GCT,CTC,AGG,AGG,AGA,GAG,ACG,AGT,TCG,AGA,GAT,ACG,ACA,AGG,TTC,AGA,ACA

12. ATPMMS-12: Tm=54, 60mer

GCG,AAT,CTC,TCT,CTG,AGT,TCA,GCA,ACC,ATC,TTT,GGT,CTG,ATG,TTC,TGA,ACC,TTG,TCG,TAT

13. ATPMMS-13: Tm=54, 60mer

GAA,CTC,AGA,GAG,AGA,TTC,GCA,CAC,CTC,AAC,CTC,ACC,TTC,TCT,ATC,GGA,GGA,CAA,ATC,TCT

14. ATPMMS-14: Tm=54, 60mer

AGC,AGT,AGG,TCT,TGT,CCC,AAC,CCT,TTG,GGA,AGA,CGT,CGA,AAG,AGA,TTT,GTC,CTC,CGA,TAG

15. ATPMMS-15: Tm=54, 60mer

TTG,GGA,CAA,GAC,CTA,CTG,CTT,GCA,GTA,CCT,TGA,GGA,CTT,CTC,TGA,GAT,CCA,CTT,CTT,CGG

16. ATPMMS-16: Tm=54, 60mer

GTA,GAT,TTC,GTA,GTC,GTT,TCC,ACC,CTC,GTA,GGT,CTT,GTC,ACC,GAA,GAA,GTG,GAT,CTC,AGA

17. ATPMMS-17: Tm=54, 60mer

GAA,ACG,ACT,ACG,AAA,TCT,ACG,AGT,CTC,CAA,AGA,CCA,TCG,GAC,ACT,CTG,TTA,CCT,CTC,CAG

18. ATPMMS-18: Tm=54, 61mer

TTA,AGA,CAT,GAA,GAG,AGC,CTT,GCA,CTT,TGC,AAC,AGT,GTC,GTC,TGG,AGA,GGT,AAC,AGA,GTG,T

***AtVTC1S:***

ATGAAGGCACTCATCCTCGTTGGAGGATTCGGAACCAGACTCAGACCACTCACTCTCTCCTTCCCAAAGCCACTCGTTGACTTCGCTAACAAGCCAATGATCCTCCACCAGATCGAGGCTCTCAAGGCAGTCGGAGTTGACGAGGTTGTTCTCGCTATCAACTACCAGCCAGAGGTTATGCTCAACTTCCTCAAGGACTTCGAGACCAAGCTCGAAATCAAGATCACCTGCTCCCAAGAGACTGAGCCACTCGGAACCGCTGGACCACTCGCTCTCGCTAGAGACAAGCTCCTCGACGGATCTGGAGAGCCATTCTTCGTTCTCAACTCCGACGTTATCTCTGAGTACCCACTCAAGGAGATGCTTGAGTTCCACAAGTCTCACGGTGGAGAGGCATCCATCATGGTCACTAAGGTTGACGAGCCATCCAAGTACGGAGTTGTTGTCATGGAAGAGTCCACTGGAAGAGTCGAGAAGTTCGTCGAGAAGCCAAAGCTCTACGTTGGCAACAAGATCAACGCTGGAATCTACCTCCTCAACCCATCTGTTCTCGACAAGATCGAACTCAGACCAACCTCCATCGAGAAGGAGACCTTCCCAAAGATCGCAGCAGCACAAGGACTCTACGCAATGGTTCTCCCAGGATTCTGGATGGACATTGGACAGCCTAGAGACTACATCACTGGTCTCAGACTCTACTTGGACTCCCTCAGAAAGAAGTCTCCTGCCAAGTTGACCTCTGGACCACACATCGTTGGAAACGTTCTCGTTGACGAGACTGCTACCATCGGTGAAGGATGCTTGATCGGACCAGACGTTGCTATCGGTCCAGGATGCATCGTTGAGTCTGGAGTCAGACTCTCCAGATGCACCGTCATGAGAGGAGTCAGAATCAAGAAGCACGCTTGCATCTCTTCTTCCATCATCGGATGGCACTCCACCGTCGGTCAGTGGGCTAGAATCGAGAACATGACCATCCTTGGTGAGGATGTTCACGTTTCTGACGAAATCTACTCCAACGGAGGAGTTGTTCTCCCACACAAGGAGATCAAGTCCAACATCCTCAAGCCAGAGATCGTTATGTAA

1. ATVTC1S-1: Tm=54, 60mer

ATG,AAG,GCA,CTC,ATC,CTC,GTT,GGA,GGA,TTC,GGA,ACC,AGA,CTC,AGA,CCA,CTC,ACT,CTC,TCC

2. ATVTC1S-2: Tm=54, 60mer

TCA,TTG,GCT,TGT,TAG,CGA,AGT,CAA,CGA,GTG,GCT,TTG,GGA,AGG,AGA,GAG,TGA,GTG,GTC,TGA

3. ATVTC1S-3: Tm=54, 60mer

CTT,CGC,TAA,CAA,GCC,AAT,GAT,CCT,CCA,CCA,GAT,CGA,GGC,TCT,CAA,GGC,AGT,CGG,AGT,TGA

4. ATVTC1S-4: Tm=54, 60mer

CAT,AAC,CTC,TGG,CTG,GTA,GTT,GAT,AGC,GAG,AAC,AAC,CTC,GTC,AAC,TCC,GAC,TGC,CTT,GAG

5. ATVTC1S-5: Tm=54, 60mer

ACT,ACC,AGC,CAG,AGG,TTA,TGC,TCA,ACT,TCC,TCA,AGG,ACT,TCG,AGA,CCA,AGC,TCG,AAA,TCA

6. ATVTC1S-6: Tm=54, 60mer

GCG,GTT,CCG,AGT,GGC,TCA,GTC,TCT,TGG,GAG,CAG,GTG,ATC,TTG,ATT,TCG,AGC,TTG,GTC,TCG

7. ATVTC1S-7: Tm=54, 60mer

ACT,GAG,CCA,CTC,GGA,ACC,GCT,GGA,CCA,CTC,GCT,CTC,GCT,AGA,GAC,AAG,CTC,CTC,GAC,GGA

8. ATVTC1S-8: Tm=54, 60mer

AGA,TAA,CGT,CGG,AGT,TGA,GAA,CGA,AGA,ATG,GCT,CTC,CAG,ATC,CGT,CGA,GGA,GCT,TGT,CTC

9. ATVTC1S-9: Tm=54, 60mer

TCT,CAA,CTC,CGA,CGT,TAT,CTC,TGA,GTA,CCC,ACT,CAA,GGA,GAT,GCT,TGA,GTT,CCA,CAA,GTC

10. ATVTC1S-10: Tm=54, 60mer

GTC,AAC,CTT,AGT,GAC,CAT,GAT,GGA,TGC,CTC,TCC,ACC,GTG,AGA,CTT,GTG,GAA,CTC,AAG,CAT

11. ATVTC1S-11: Tm=54, 60mer

TCA,TGG,TCA,CTA,AGG,TTG,ACG,AGC,CAT,CCA,AGT,ACG,GAG,TTG,TTG,TCA,TGG,AAG,AGT,CCA

12. ATVTC1S-12: Tm=54, 60mer

TAG,AGC,TTT,GGC,TTC,TCG,ACG,AAC,TTC,TCG,ACT,CTT,CCA,GTG,GAC,TCT,TCC,ATG,ACA,ACA

13. ATVTC1S-13: Tm=54, 60mer

GTC,GAG,AAG,CCA,AAG,CTC,TAC,GTT,GGC,AAC,AAG,ATC,AAC,GCT,GGA,ATC,TAC,CTC,CTC,AAC

14. ATVTC1S-14: Tm=54, 60mer

TGG,AGG,TTG,GTC,TGA,GTT,CGA,TCT,TGT,CGA,GAA,CAG,ATG,GGT,TGA,GGA,GGT,AGA,TTC,CAG

15. ATVTC1S-15: Tm=54, 60mer

CGA,ACT,CAG,ACC,AAC,CTC,CAT,CGA,GAA,GGA,GAC,CTT,CCC,AAA,GAT,CGC,AGC,AGC,ACA,AGG

16. ATVTC1S-16: Tm=54, 60mer

AAT,GTC,CAT,CCA,GAA,TCC,TGG,GAG,AAC,CAT,TGC,GTA,GAG,TCC,TTG,TGC,TGC,TGC,GAT,CTT

17. ATVTC1S-17: Tm=54, 60mer

CAG,GAT,TCT,GGA,TGG,ACA,TTG,GAC,AGC,CTA,GAG,ACT,ACA,TCA,CTG,GTC,TCA,GAC,TCT,ACT

18. ATVTC1S-18: Tm=54, 60mer

GAG,GTC,AAC,TTG,GCA,GGA,GAC,TTC,TTT,CTG,AGG,GAG,TCC,AAG,TAG,AGT,CTG,AGA,CCA,GTG

19. ATVTC1S-19: Tm=54, 60mer

TCT,CCT,GCC,AAG,TTG,ACC,TCT,GGA,CCA,CAC,ATC,GTT,GGA,AAC,GTT,CTC,GTT,GAC,GAG,ACT

20. ATVTC1S-20: Tm=54, 60mer

CAA,CGT,CTG,GTC,CGA,TCA,AGC,ATC,CTT,CAC,CGA,TGG,TAG,CAG,TCT,CGT,CAA,CGA,GAA,CGT

21. ATVTC1S-21: Tm=54, 60mer

CTT,GAT,CGG,ACC,AGA,CGT,TGC,TAT,CGG,TCC,AGG,ATG,CAT,CGT,TGA,GTC,TGG,AGT,CAG,ACT

22. ATVTC1S-22: Tm=54, 60mer

CTT,CTT,GAT,TCT,GAC,TCC,TCT,CAT,GAC,GGT,GCA,TCT,GGA,GAG,TCT,GAC,TCC,AGA,CTC,AAC

23. ATVTC1S-23: Tm=54, 60mer

GAG,GAG,TCA,GAA,TCA,AGA,AGC,ACG,CTT,GCA,TCT,CTT,CTT,CCA,TCA,TCG,GAT,GGC,ACT,CCA

24. ATVTC1S-24: Tm=54, 60mer

AGG,ATG,GTC,ATG,TTC,TCG,ATT,CTA,GCC,CAC,TGA,CCG,ACG,GTG,GAG,TGC,CAT,CCG,ATG,ATG

25. ATVTC1S-25: Tm=54, 60mer

ATC,GAG,AAC,ATG,ACC,ATC,CTT,GGT,GAG,GAT,GTT,CAC,GTT,TCT,GAC,GAA,ATC,TAC,TCC,AAC

26. ATVTC1S-26: Tm=54, 60mer

TGT,TGG,ACT,TGA,TCT,CCT,TGT,GTG,GGA,GAA,CAA,CTC,CTC,CGT,TGG,AGT,AGA,TTT,CGT,CAG

27. ATVTC1S-27: Tm=54, 46mer

TTA,CAT,AAC,GAT,CTC,TGG,CTT,GAG,GAT,GTT,GGA,CTT,GAT,CTC,CTT,G

***AtGMES:***

ATGGGAACTACCAACGGAACCGACTACGGAGCATACACCTACAAGGAGCTTGAGAGAGAGCAGTACTGGCCATCTGAGAACCTCAAAATCTCCATCACCGGAGCTGGAGGATTCATCGCATCTCACATCGCTAGAAGACTCAAGCACGAGGGTCACTACGTCATCGCATCTGACTGGAAGAAGAACGAGCACATGACCGAGGACATGTTCTGCGACGAGTTCCACCTCGTTGACCTCAGAGTCATGGAGAACTGTCTCAAGGTTACTGAAGGAGTTGACCACGTCTTCAACTTGGCTGCTGACATGGGTGGAATGGGTTTCATCCAGTCCAACCACTCTGTCATCATGTACAACAACACCATGATCTCCTTCAACATGATCGAGGCTGCTAGAATCAACGGAATCAAGAGATTCTTCTACGCTTCCTCTGCTTGCATCTACCCAGAGTTCAAGCAGCTTGAGACCACCAACGTCTCTCTCAAGGAGTCCGACGCTTGGCCAGCAGAGCCTCAAGATGCTTACGGACTTGAGAAGCTCGCTACCGAGGAGTTGTGCAAGCACTACAACAAGGACTTCGGTATCGAGTGCAGAATCGGAAGATTCCACAACATCTACGGTCCTTTCGGAACCTGGAAGGGTGGAAGAGAGAAGGCTCCAGCTGCATTCTGTAGAAAGGCTCAGACCTCCACTGACAGATTCGAGATGTGGGGAGACGGACTCCAGACCAGATCCTTCACCTTCATCGACGAGTGCGTCGAAGGTGTTCTCAGATTGACCAAGTCCGACTTCAGAGAGCCAGTCAACATCGGATCTGACGAGATGGTCTCTATGAACGAGATGGCTGAGATGGTTCTCTCCTTCGAGGAGAAGAAGCTCCCAATCCACCACATCCCAGGACCAGAAGGTGTCAGAGGTAGAAACTCCGACAACAACCTCATCAAGGAGAAGCTCGGTTGGGCTCCTAACATGAGACTCAAGGAGGGACTCAGAATCACCTACTTCTGGATCAAGGAACAGATCGAGAAGGAGAAGGCAAAGGGATCTGACGTCTCTCTCTACGGATCTTCCAAGGTTGTTGGAACTCAGGCACCTGTTCAGCTCGGATCTCTCAGAGCAGCTGACGGAAAGGAGTAA

1. ATGMES-1: Tm=54, 60mer

ATG,GGA,ACT,ACC,AAC,GGA,ACC,GAC,TAC,GGA,GCA,TAC,ACC,TAC,AAG,GAG,CTT,GAG,AGA,GAG

2. ATGMES-2: Tm=54, 60mer

CGG,TGA,TGG,AGA,TTT,TGA,GGT,TCT,CAG,ATG,GCC,AGT,ACT,GCT,CTC,TCT,CAA,GCT,CCT,TGT

3. ATGMES-3: Tm=54, 60mer

CCT,CAA,AAT,CTC,CAT,CAC,CGG,AGC,TGG,AGG,ATT,CAT,CGC,ATC,TCA,CAT,CGC,TAG,AAG,ACT

4. ATGMES-4: Tm=54, 60mer

CTT,CCA,GTC,AGA,TGC,GAT,GAC,GTA,GTG,ACC,CTC,GTG,CTT,GAG,TCT,TCT,AGC,GAT,GTG,AGA

5. ATGMES-5: Tm=54, 60mer

TCA,TCG,CAT,CTG,ACT,GGA,AGA,AGA,ACG,AGC,ACA,TGA,CCG,AGG,ACA,TGT,TCT,GCG,ACG,AGT

6. ATGMES-6: Tm=54, 60mer

TTG,AGA,CAG,TTC,TCC,ATG,ACT,CTG,AGG,TCA,ACG,AGG,TGG,AAC,TCG,TCG,CAG,AAC,ATG,TCC

7. ATGMES-7: Tm=54, 60mer

GTC,ATG,GAG,AAC,TGT,CTC,AAG,GTT,ACT,GAA,GGA,GTT,GAC,CAC,GTC,TTC,AAC,TTG,GCT,GCT

8. ATGMES-8: Tm=54, 60mer

CAG,AGT,GGT,TGG,ACT,GGA,TGA,AAC,CCA,TTC,CAC,CCA,TGT,CAG,CAG,CCA,AGT,TGA,AGA,CGT

9. ATGMES-9: Tm=54, 60mer

CAT,CCA,GTC,CAA,CCA,CTC,TGT,CAT,CAT,GTA,CAA,CAA,CAC,CAT,GAT,CTC,CTT,CAA,CAT,GAT

10. ATGMES-10: Tm=54, 60mer

GTA,GAA,GAA,TCT,CTT,GAT,TCC,GTT,GAT,TCT,AGC,AGC,CTC,GAT,CAT,GTT,GAA,GGA,GAT,CAT

11. ATGMES-11: Tm=54, 60mer

GAA,TCA,AGA,GAT,TCT,TCT,ACG,CTT,CCT,CTG,CTT,GCA,TCT,ACC,CAG,AGT,TCA,AGC,AGC,TTG

12. ATGMES-12: Tm=54, 60mer

GGC,CAA,GCG,TCG,GAC,TCC,TTG,AGA,GAG,ACG,TTG,GTG,GTC,TCA,AGC,TGC,TTG,AAC,TCT,GGG

13. ATGMES-13: Tm=54, 60mer

AAG,GAG,TCC,GAC,GCT,TGG,CCA,GCA,GAG,CCT,CAA,GAT,GCT,TAC,GGA,CTT,GAG,AAG,CTC,GCT

14. ATGMES-14: Tm=54, 60mer

TAC,CGA,AGT,CCT,TGT,TGT,AGT,GCT,TGC,ACA,ACT,CCT,CGG,TAG,CGA,GCT,TCT,CAA,GTC,CGT

15. ATGMES-15: Tm=54, 60mer

CTA,CAA,CAA,GGA,CTT,CGG,TAT,CGA,GTG,CAG,AAT,CGG,AAG,ATT,CCA,CAA,CAT,CTA,CGG,TCC

16. ATGMES-16: Tm=54, 60mer

AGC,TGG,AGC,CTT,CTC,TCT,TCC,ACC,CTT,CCA,GGT,TCC,GAA,AGG,ACC,GTA,GAT,GTT,GTG,GAA

17. ATGMES-17: Tm=54, 60mer

GAA,GAG,AGA,AGG,CTC,CAG,CTG,CAT,TCT,GTA,GAA,AGG,CTC,AGA,CCT,CCA,CTG,ACA,GAT,TCG

18. ATGMES-18: Tm=54, 60mer

AAG,GTG,AAG,GAT,CTG,GTC,TGG,AGT,CCG,TCT,CCC,CAC,ATC,TCG,AAT,CTG,TCA,GTG,GAG,GTC

19. ATGMES-19: Tm=54, 60mer

CAG,ACC,AGA,TCC,TTC,ACC,TTC,ATC,GAC,GAG,TGC,GTC,GAA,GGT,GTT,CTC,AGA,TTG,ACC,AAG

20. ATGMES-20: Tm=54, 60mer

TCT,CGT,CAG,ATC,CGA,TGT,TGA,CTG,GCT,CTC,TGA,AGT,CGG,ACT,TGG,TCA,ATC,TGA,GAA,CAC

21. ATGMES-21: Tm=54, 60mer

CAA,CAT,CGG,ATC,TGA,CGA,GAT,GGT,CTC,TAT,GAA,CGA,GAT,GGC,TGA,GAT,GGT,TCT,CTC,CTT

22. ATGMES-22: Tm=54, 60mer

TGG,TCC,TGG,GAT,GTG,GTG,GAT,TGG,GAG,CTT,CTT,CTC,CTC,GAA,GGA,GAG,AAC,CAT,CTC,AGC

23. ATGMES-23: Tm=54, 60mer

TCC,ACC,ACA,TCC,CAG,GAC,CAG,AAG,GTG,TCA,GAG,GTA,GAA,ACT,CCG,ACA,ACA,ACC,TCA,TCA

24. ATGMES-24: Tm=54, 60mer

TCC,TTG,AGT,CTC,ATG,TTA,GGA,GCC,CAA,CCG,AGC,TTC,TCC,TTG,ATG,AGG,TTG,TTG,TCG,GAG

25. ATGMES-25: Tm=54, 60mer

CCT,AAC,ATG,AGA,CTC,AAG,GAG,GGA,CTC,AGA,ATC,ACC,TAC,TTC,TGG,ATC,AAG,GAA,CAG,ATC

26. ATGMES-26: Tm=54, 60mer

CGT,AGA,GAG,AGA,CGT,CAG,ATC,CCT,TTG,CCT,TCT,CCT,TCT,CGA,TCT,GTT,CCT,TGA,TCC,AGA

27. ATGMES-27: Tm=54, 60mer

ATC,TGA,CGT,CTC,TCT,CTA,CGG,ATC,TTC,CAA,GGT,TGT,TGG,AAC,TCA,GGC,ACC,TGT,TCA,GCT

28. ATGMES-28: Tm=54, 54mer

TTA,CTC,CTT,TCC,GTC,AGC,TGC,TCT,GAG,AGA,TCC,GAG,CTG,AAC,AGG,TGC,CTG,AGT

***AtVTC2S:***  ATGTTGAAGATCAAGAGAGTTCCAACCGTTGTTTCCAACTACCAGAAGGACGACGGAGCAGAGGACCCAGTTGGATGTGGAAGAAACTGTCTTGGTGCCTGTTGCCTCAACGGAGCTAGACTCCCACTCTACGCATGTAAGAACCTCGTCAAGTCTGGAGAGAAGCTCGTCATCTCTCACGAGGCTATCGAGCCACCAGTTGCATTCCTCGAATCCCTCGTTCTCGGAGAGTGGGAGGACAGATTCCAGAGAGGACTCTTCAGATACGACGTTACTGCATGTGAGACCAAGGTTATCCCAGGAAAGTACGGATTCGTTGCTCAGCTCAACGAGGGTAGACACCTCAAGAAGAGACCAACCGAGTTCAGAGTTGACAAGGTCTTGCAGTCCTTCGACGGTTCCAAGTTCAACTTCACCAAGGTTGGACAGGAGGAGTTGCTCTTCCAGTTCGAAGCAGGTGAGGACGCACAAGTTCAGTTCTTCCCTTGTATGCCAATCGATCCTGAGAACTCTCCATCTGTTGTTGCAATCAACGTCTCTCCAATCGAGTACGGACACGTTCTCCTCATCCCTAGAGTTCTCGACTGCCTCCCACAGAGAATCGATCACAAGTCTCTCCTCCTTGCAGTTCACATGGCTGCTGAGGCTGCTAACCCATACTTCAGACTCGGTTACAACTCTCTCGGTGCTTTCGCTACCATCAACCACCTCCACTTCCAGGCTTACTACCTCGCTATGCCATTCCCACTTGAGAAGGCTCCAACCAAGAAGATCACCACCACTGTTTCTGGTGTCAAAATCTCTGAGCTTCTCTCCTACCCTGTCAGATCCCTCCTCTTCGAAGGTGGATCTTCCATGCAGGAACTCTCTGACACCGTTTCTGACTGCTGCGTCTGCCTCCAGAACAACAACATCCCATTCAACATCCTCATCTCTGACTGTGGAAGACAAATCTTCCTCATGCCACAGTGCTACGCAGAGAAGCAGGCTCTCGGAGAAGTTTCTCCTGAGGTCTTGGAGACCCAAGTCAACCCAGCTGTCTGGGAAATCTCTGGACACATGGTTCTCAAGAGAAAGGAGGACTACGAGGGTGCTTCCGAGGACAACGCATGGAGACTCCTCGCAGAAGCATCTCTCTCCGAGGAGAGATTCAAGGAGGTTACTGCTCTCGCATTCGAGGCTATCGGATGCTCCAACCAAGAGGAGGACCTCGAAGGAACCATCGTTCACCAGCAGAACTCCTCTGGCAACGTCAACCAGAAGTCCAACAGAACTCATGGAGGACCAATCACCAACGGAACTGCTGCTGAGTGTCTCGTTCTCCAGTAA

1. ATVTC2S-1: Tm=54, 60mer

ATG,TTG,AAG,ATC,AAG,AGA,GTT,CCA,ACC,GTT,GTT,TCC,AAC,TAC,CAG,AAG,GAC,GAC,GGA,GCA

2. ATVTC2S-2: Tm=54, 60mer

AGG,CAC,CAA,GAC,AGT,TTC,TTC,CAC,ATC,CAA,CTG,GGT,CCT,CTG,CTC,CGT,CGT,CCT,TCT,GGT

3. ATVTC2S-3: Tm=54, 60mer

AAG,AAA,CTG,TCT,TGG,TGC,CTG,TTG,CCT,CAA,CGG,AGC,TAG,ACT,CCC,ACT,CTA,CGC,ATG,TAA

4. ATVTC2S-4: Tm=54, 60mer

GTG,AGA,GAT,GAC,GAG,CTT,CTC,TCC,AGA,CTT,GAC,GAG,GTT,CTT,ACA,TGC,GTA,GAG,TGG,GAG

5. ATVTC2S-5: Tm=54, 60mer

AGA,AGC,TCG,TCA,TCT,CTC,ACG,AGG,CTA,TCG,AGC,CAC,CAG,TTG,CAT,TCC,TCG,AAT,CCC,TCG

6. ATVTC2S-6: Tm=54, 60mer

AAG,AGT,CCT,CTC,TGG,AAT,CTG,TCC,TCC,CAC,TCT,CCG,AGA,ACG,AGG,GAT,TCG,AGG,AAT,GCA

7. ATVTC2S-7: Tm=54, 60mer

AGA,TTC,CAG,AGA,GGA,CTC,TTC,AGA,TAC,GAC,GTT,ACT,GCA,TGT,GAG,ACC,AAG,GTT,ATC,CCA

8. ATVTC2S-8: Tm=54, 60mer

GTC,TAC,CCT,CGT,TGA,GCT,GAG,CAA,CGA,ATC,CGT,ACT,TTC,CTG,GGA,TAA,CCT,TGG,TCT,CAC

9. ATVTC2S-9: Tm=54, 60mer

TCA,GCT,CAA,CGA,GGG,TAG,ACA,CCT,CAA,GAA,GAG,ACC,AAC,CGA,GTT,CAG,AGT,TGA,CAA,GGT

10. ATVTC2S-10: Tm=54, 60mer

CTT,GGT,GAA,GTT,GAA,CTT,GGA,ACC,GTC,GAA,GGA,CTG,CAA,GAC,CTT,GTC,AAC,TCT,GAA,CTC

11. ATVTC2S-11: Tm=54, 60mer

CCA,AGT,TCA,ACT,TCA,CCA,AGG,TTG,GAC,AGG,AGG,AGT,TGC,TCT,TCC,AGT,TCG,AAG,CAG,GTG

12. ATVTC2S-12: Tm=54, 60mer

TCG,ATT,GGC,ATA,CAA,GGG,AAG,AAC,TGA,ACT,TGT,GCG,TCC,TCA,CCT,GCT,TCG,AAC,TGG,AAG

13. ATVTC2S-13: Tm=54, 60mer

TTC,CCT,TGT,ATG,CCA,ATC,GAT,CCT,GAG,AAC,TCT,CCA,TCT,GTT,GTT,GCA,ATC,AAC,GTC,TCT

14. ATVTC2S-14: Tm=54, 60mer

GAA,CTC,TAG,GGA,TGA,GGA,GAA,CGT,GTC,CGT,ACT,CGA,TTG,GAG,AGA,CGT,TGA,TTG,CAA,CAA

15. ATVTC2S-15: Tm=54, 60mer

TCT,CCT,CAT,CCC,TAG,AGT,TCT,CGA,CTG,CCT,CCC,ACA,GAG,AAT,CGA,TCA,CAA,GTC,TCT,CCT

16. ATVTC2S-16: Tm=54, 60mer

GTA,TGG,GTT,AGC,AGC,CTC,AGC,AGC,CAT,GTG,AAC,TGC,AAG,GAG,GAG,AGA,CTT,GTG,ATC,GAT

17. ATVTC2S-17: Tm=54, 60mer

CTG,AGG,CTG,CTA,ACC,CAT,ACT,TCA,GAC,TCG,GTT,ACA,ACT,CTC,TCG,GTG,CTT,TCG,CTA,CCA

18. ATVTC2S-18: Tm=54, 60mer

GGC,ATA,GCG,AGG,TAG,TAA,GCC,TGG,AAG,TGG,AGG,TGG,TTG,ATG,GTA,GCG,AAA,GCA,CCG,AGA

19. ATVTC2S-19: Tm=54, 60mer

GCT,TAC,TAC,CTC,GCT,ATG,CCA,TTC,CCA,CTT,GAG,AAG,GCT,CCA,ACC,AAG,AAG,ATC,ACC,ACC

20. ATVTC2S-20: Tm=54, 60mer

GGT,AGG,AGA,GAA,GCT,CAG,AGA,TTT,TGA,CAC,CAG,AAA,CAG,TGG,TGG,TGA,TCT,TCT,TGG,TTG

21. ATVTC2S-21: Tm=54, 60mer

CTC,TGA,GCT,TCT,CTC,CTA,CCC,TGT,CAG,ATC,CCT,CCT,CTT,CGA,AGG,TGG,ATC,TTC,CAT,GCA

22. ATVTC2S-22: Tm=54, 60mer

GAG,GCA,GAC,GCA,GCA,GTC,AGA,AAC,GGT,GTC,AGA,GAG,TTC,CTG,CAT,GGA,AGA,TCC,ACC,TTC

23. ATVTC2S-23: Tm=54, 60mer

CTG,ACT,GCT,GCG,TCT,GCC,TCC,AGA,ACA,ACA,ACA,TCC,CAT,TCA,ACA,TCC,TCA,TCT,CTG,ACT

24. ATVTC2S-24: Tm=54, 60mer

TCT,GCG,TAG,CAC,TGT,GGC,ATG,AGG,AAG,ATT,TGT,CTT,CCA,CAG,TCA,GAG,ATG,AGG,ATG,TTG

25. ATVTC2S-25: Tm=54, 60mer

ATG,CCA,CAG,TGC,TAC,GCA,GAG,AAG,CAG,GCT,CTC,GGA,GAA,GTT,TCT,CCT,GAG,GTC,TTG,GAG

26. ATVTC2S-26: Tm=54, 60mer

TGT,GTC,CAG,AGA,TTT,CCC,AGA,CAG,CTG,GGT,TGA,CTT,GGG,TCT,CCA,AGA,CCT,CAG,GAG,AAA

27. ATVTC2S-27: Tm=54, 60mer

CTG,GGA,AAT,CTC,TGG,ACA,CAT,GGT,TCT,CAA,GAG,AAA,GGA,GGA,CTA,CGA,GGG,TGC,TTC,CGA

28. ATVTC2S-28: Tm=54, 60mer

GGA,GAG,AGA,TGC,TTC,TGC,GAG,GAG,TCT,CCA,TGC,GTT,GTC,CTC,GGA,AGC,ACC,CTC,GTA,GTC

29. ATVTC2S-29: Tm=54, 60mer

TCG,CAG,AAG,CAT,CTC,TCT,CCG,AGG,AGA,GAT,TCA,AGG,AGG,TTA,CTG,CTC,TCG,CAT,TCG,AGG

30. ATVTC2S-30: Tm=54, 60mer

GTT,CCT,TCG,AGG,TCC,TCC,TCT,TGG,TTG,GAG,CAT,CCG,ATA,GCC,TCG,AAT,GCG,AGA,GCA,GTA

31. ATVTC2S-31: Tm=54, 60mer

GAG,GAG,GAC,CTC,GAA,GGA,ACC,ATC,GTT,CAC,CAG,CAG,AAC,TCC,TCT,GGC,AAC,GTC,AAC,CAG

32. ATVTC2S-32: Tm=54, 60mer

TTC,CGT,TGG,TGA,TTG,GTC,CTC,CAT,GAG,TTC,TGT,TGG,ACT,TCT,GGT,TGA,CGT,TGC,CAG,AGG

33. ATVTC2S-33: Tm=54, 49mer

TTA,CTG,GAG,AAC,GAG,ACA,CTC,AGC,AGC,AGT,TCC,GTT,GGT,GAT,TGG,TCC,T

***AtVTC4S:*** ATGGCAGACAACGACCAGTTCTTGGCTGCTGCCATCGACGCTGCTAAGAAGGCTGGACAGATCATCAGAAAGGGATTCTACGAGACCAAGCACGTCGAACACAAGGGACAGGTTGACCTCGTCACTGAGACTGACAAGGGATGCGAGGAGCTTGTCTTCAACCACCTCAAGCAGCTCTTCCCTAACCACAAGTTCATCGGAGAGGAGACCACTGCTGCATTCGGTGTTACCGAACTCACCGACGAACCAACCTGGATCGTTGACCCACTCGACGGAACCACCAACTTCGTTCACGGATTCCCATTCGTTTGCGTTTCCATCGGACTCACCATCGGAAAGGTCCCTGTTGTTGGAGTTGTCTACAACCCTATCATGGAAGAACTCTTCACTGGTGTTCAGGGTAAGGGAGCATTCTTGAACGGCAAGAGAATCAAGGTTTCCGCTCAGTCTGAACTCCTCACTGCTCTCCTCGTCACCGAGGCTGGAACCAAGAGAGACAAGGCTACCTTGGACGACACCACCAACAGAATCAACTCCCTCCTCACCAAGGTTAGATCCCTCAGAATGTCTGGTTCCTGTGCACTCGACCTCTGTGGAGTTGCATGTGGAAGAGTTGACATCTTCTACGAACTCGGTTTCGGTGGTCCTTGGGACATCGCAGCAGGCATCGTTATCGTCAAGGAAGCTGGTGGACTCATCTTCGACCCATCTGGTAAGGACCTCGACATCACCTCCCAGAGAATCGCAGCTTCCAACGCATCTCTCAAGGAGTTGTTCGCTGAGGCACTCAGACTCACTGGAGCATAA

1. ATVTC4S-1: Tm=54, 60mer

ATG,GCA,GAC,AAC,GAC,CAG,TTC,TTG,GCT,GCT,GCC,ATC,GAC,GCT,GCT,AAG,AAG,GCT,GGA,CAG

2. ATVTC4S-2: Tm=54, 60mer

GTT,CGA,CGT,GCT,TGG,TCT,CGT,AGA,ATC,CCT,TTC,TGA,TGA,TCT,GTC,CAG,CCT,TCT,TAG,CAG

3. ATVTC4S-3: Tm=54, 60mer

CGA,GAC,CAA,GCA,CGT,CGA,ACA,CAA,GGG,ACA,GGT,TGA,CCT,CGT,CAC,TGA,GAC,TGA,CAA,GGG

4. ATVTC4S-4: Tm=54, 60mer

GAA,GAG,CTG,CTT,GAG,GTG,GTT,GAA,GAC,AAG,CTC,CTC,GCA,TCC,CTT,GTC,AGT,CTC,AGT,GAC

5. ATVTC4S-5: Tm=54, 60mer

ACC,ACC,TCA,AGC,AGC,TCT,TCC,CTA,ACC,ACA,AGT,TCA,TCG,GAG,AGG,AGA,CCA,CTG,CTG,CAT

6. ATVTC4S-6: Tm=54, 60mer

ACG,ATC,CAG,GTT,GGT,TCG,TCG,GTG,AGT,TCG,GTA,ACA,CCG,AAT,GCA,GCA,GTG,GTC,TCC,TCT

7. ATVTC4S-7: Tm=54, 60mer

GAC,GAA,CCA,ACC,TGG,ATC,GTT,GAC,CCA,CTC,GAC,GGA,ACC,ACC,AAC,TTC,GTT,CAC,GGA,TTC

8. ATVTC4S-8: Tm=54, 60mer

CCT,TTC,CGA,TGG,TGA,GTC,CGA,TGG,AAA,CGC,AAA,CGA,ATG,GGA,ATC,CGT,GAA,CGA,AGT,TGG

9. ATVTC4S-9: Tm=54, 60mer

CGG,ACT,CAC,CAT,CGG,AAA,GGT,CCC,TGT,TGT,TGG,AGT,TGT,CTA,CAA,CCC,TAT,CAT,GGA,AGA

10. ATVTC4S-10: Tm=54, 60mer

GTT,CAA,GAA,TGC,TCC,CTT,ACC,CTG,AAC,ACC,AGT,GAA,GAG,TTC,TTC,CAT,GAT,AGG,GTT,GTA

11. ATVTC4S-11: Tm=54, 60mer

GTA,AGG,GAG,CAT,TCT,TGA,ACG,GCA,AGA,GAA,TCA,AGG,TTT,CCG,CTC,AGT,CTG,AAC,TCC,TCA

12. ATVTC4S-12: Tm=54, 60mer

TTG,TCT,CTC,TTG,GTT,CCA,GCC,TCG,GTG,ACG,AGG,AGA,GCA,GTG,AGG,AGT,TCA,GAC,TGA,GCG

13. ATVTC4S-13: Tm=54, 60mer

GCT,GGA,ACC,AAG,AGA,GAC,AAG,GCT,ACC,TTG,GAC,GAC,ACC,ACC,AAC,AGA,ATC,AAC,TCC,CTC

14. ATVTC4S-14: Tm=54, 60mer

CAC,AGG,AAC,CAG,ACA,TTC,TGA,GGG,ATC,TAA,CCT,TGG,TGA,GGA,GGG,AGT,TGA,TTC,TGT,TGG

15. ATVTC4S-15: Tm=54, 60mer

CAG,AAT,GTC,TGG,TTC,CTG,TGC,ACT,CGA,CCT,CTG,TGG,AGT,TGC,ATG,TGG,AAG,AGT,TGA,CAT

16. ATVTC4S-16: Tm=54, 60mer

TGC,GAT,GTC,CCA,AGG,ACC,ACC,GAA,ACC,GAG,TTC,GTA,GAA,GAT,GTC,AAC,TCT,TCC,ACA,TGC

17. ATVTC4S-17: Tm=54, 60mer

GTG,GTC,CTT,GGG,ACA,TCG,CAG,CAG,GCA,TCG,TTA,TCG,TCA,AGG,AAG,CTG,GTG,GAC,TCA,TCT

18. ATVTC4S-18: Tm=54, 60mer

CTC,TGG,GAG,GTG,ATG,TCG,AGG,TCC,TTA,CCA,GAT,GGG,TCG,AAG,ATG,AGT,CCA,CCA,GCT,TCC

19. ATVTC4S-19: Tm=54, 60mer

CTC,GAC,ATC,ACC,TCC,CAG,AGA,ATC,GCA,GCT,TCC,AAC,GCA,TCT,CTC,AAG,GAG,TTG,TTC,GCT

20. ATVTC4S-20: Tm=54, 47mer

TTA,TGC,TCC,AGT,GAG,TCT,GAG,TGC,CTC,AGC,GAA,CAA,CTC,CTT,GAG,AG

***AtGALDHS:***

ATGACTAAGATCGAACTCAGAGCACTTGGAAACACTGGACTCAAGGTTTCTGCTGTCGGATTCGGTGCATCTCCACTCGGATCTGTCTTCGGACCAGTTGCTGAAGACGACGCTGTTGCAACTGTCAGAGAGGCTTTCAGACTCGGTATCAACTTCTTCGACACCTCTCCATACTACGGAGGAACCCTCTCTGAGAAGATGCTCGGTAAGGGACTCAAGGCTCTCCAAGTTCCTAGATCCGACTACATCGTTGCTACCAAGTGTGGTAGATACAAAGAGGGTTTCGACTTCTCTGCTGAGAGAGTCAGAAAGTCCATCGACGAGTCCTTGGAGAGACTCCAGCTCGACTACGTTGACATCCTCCACTGCCACGACATCGAGTTTGGATCTCTCGACCAGATCGTCTCTGAGACCATCCCTGCTCTCCAGAAGCTCAAGCAGGAGGGTAAGACCAGATTCATCGGTATCACTGGTCTCCCATTGGACATCTTCACCTACGTTCTCGACAGAGTTCCACCAGGAACTGTTGACGTTATCTTGTCTTACTGTCACTACGGTGTCAACGACTCTACCTTGCTCGACTTGCTCCCATACTTGAAGTCTAAGGGTGTTGGTGTCATCTCTGCTTCTCCATTGGCAATGGGACTCCTCACCGAACAAGGACCACCTGAGTGGCACCCTGCTTCTCCTGAACTCAAGTCCGCATCCAAGGCTGCTGTTGCTCACTGCAAGTCTAAGGGTAAGAAGATCACCAAGTTGGCTCTCCAGTACTCTCTCGCAAACAAGGAAATCTCTTCTGTCTTGGTTGGAATGTCTTCTGTCTCTCAGGTTGAAGAGAACGTTGCAGCAGTCACCGAGCTTGAGTCTCTCGGTATGGACCAGGAGACTCTCTCTGAGGTCGAAGCCATCCTCGAACCAGTCAAGAACCTCACCTGGCCATCTGGAATCCACCAGAACTAA

1. ATGALDHSS-1: Tm=54, 60mer

ATG,ACT,AAG,ATC,GAA,CTC,AGA,GCA,CTT,GGA,AAC,ACT,GGA,CTC,AAG,GTT,TCT,GCT,GTC,GGA

2. ATGALDHSS-2: Tm=54, 60mer

CAA,CTG,GTC,CGA,AGA,CAG,ATC,CGA,GTG,GAG,ATG,CAC,CGA,ATC,CGA,CAG,CAG,AAA,CCT,TGA

3. ATGALDHSS-3: Tm=54, 60mer

ATC,TGT,CTT,CGG,ACC,AGT,TGC,TGA,AGA,CGA,CGC,TGT,TGC,AAC,TGT,CAG,AGA,GGC,TTT,CAG

4. ATGALDHSS-4: Tm=54, 60mer

TCC,GTA,GTA,TGG,AGA,GGT,GTC,GAA,GAA,GTT,GAT,ACC,GAG,TCT,GAA,AGC,CTC,TCT,GAC,AGT

5. ATGALDHSS-5: Tm=54, 60mer

ACA,CCT,CTC,CAT,ACT,ACG,GAG,GAA,CCC,TCT,CTG,AGA,AGA,TGC,TCG,GTA,AGG,GAC,TCA,AGG

6. ATGALDHSS-6: Tm=54, 60mer

TTG,GTA,GCA,ACG,ATG,TAG,TCG,GAT,CTA,GGA,ACT,TGG,AGA,GCC,TTG,AGT,CCC,TTA,CCG,AGC

7. ATGALDHSS-7: Tm=54, 60mer

GAC,TAC,ATC,GTT,GCT,ACC,AAG,TGT,GGT,AGA,TAC,AAA,GAG,GGT,TTC,GAC,TTC,TCT,GCT,GAG

8. ATGALDHSS-8: Tm=54, 60mer

GGA,GTC,TCT,CCA,AGG,ACT,CGT,CGA,TGG,ACT,TTC,TGA,CTC,TCT,CAG,CAG,AGA,AGT,CGA,AAC

9. ATGALDHSS-9: Tm=54, 60mer

CGA,GTC,CTT,GGA,GAG,ACT,CCA,GCT,CGA,CTA,CGT,TGA,CAT,CCT,CCA,CTG,CCA,CGA,CAT,CGA

10. ATGALDHSS-10: Tm=54, 60mer

AGG,GAT,GGT,CTC,AGA,GAC,GAT,CTG,GTC,GAG,AGA,TCC,AAA,CTC,GAT,GTC,GTG,GCA,GTG,GAG

11. ATGALDHSS-11: Tm=54, 60mer

TCG,TCT,CTG,AGA,CCA,TCC,CTG,CTC,TCC,AGA,AGC,TCA,AGC,AGG,AGG,GTA,AGA,CCA,GAT,TCA

12. ATGALDHSS-12: Tm=54, 60mer

ACG,TAG,GTG,AAG,ATG,TCC,AAT,GGG,AGA,CCA,GTG,ATA,CCG,ATG,AAT,CTG,GTC,TTA,CCC,TCC

13. ATGALDHSS-13: Tm=54, 60mer

TTG,GAC,ATC,TTC,ACC,TAC,GTT,CTC,GAC,AGA,GTT,CCA,CCA,GGA,ACT,GTT,GAC,GTT,ATC,TTG

14. ATGALDHSS-14: Tm=54, 60mer

CGA,GCA,AGG,TAG,AGT,CGT,TGA,CAC,CGT,AGT,GAC,AGT,AAG,ACA,AGA,TAA,CGT,CAA,CAG,TTC

15. ATGALDHSS-15: Tm=54, 60mer

CAA,CGA,CTC,TAC,CTT,GCT,CGA,CTT,GCT,CCC,ATA,CTT,GAA,GTC,TAA,GGG,TGT,TGG,TGT,CAT

16. ATGALDHSS-16: Tm=54, 60mer

TTG,TTC,GGT,GAG,GAG,TCC,CAT,TGC,CAA,TGG,AGA,AGC,AGA,GAT,GAC,ACC,AAC,ACC,CTT,AGA

17. ATGALDHSS-17: Tm=54, 60mer

TGG,GAC,TCC,TCA,CCG,AAC,AAG,GAC,CAC,CTG,AGT,GGC,ACC,CTG,CTT,CTC,CTG,AAC,TCA,AGT

18. ATGALDHSS-18: Tm=54, 60mer

CCC,TTA,GAC,TTG,CAG,TGA,GCA,ACA,GCA,GCC,TTG,GAT,GCG,GAC,TTG,AGT,TCA,GGA,GAA,GCA

19. ATGALDHSS-19: Tm=54, 60mer

GCT,CAC,TGC,AAG,TCT,AAG,GGT,AAG,AAG,ATC,ACC,AAG,TTG,GCT,CTC,CAG,TAC,TCT,CTC,GCA

20. ATGALDHSS-20: Tm=54, 60mer

CAG,AAG,ACA,TTC,CAA,CCA,AGA,CAG,AAG,AGA,TTT,CCT,TGT,TTG,CGA,GAG,AGT,ACT,GGA,GAG

21. ATGALDHSS-21: Tm=54, 60mer

CTT,GGT,TGG,AAT,GTC,TTC,TGT,CTC,TCA,GGT,TGA,AGA,GAA,CGT,TGC,AGC,AGT,CAC,CGA,GCT

22. ATGALDHSS-22: Tm=54, 60mer

GAC,CTC,AGA,GAG,AGT,CTC,CTG,GTC,CAT,ACC,GAG,AGA,CTC,AAG,CTC,GGT,GAC,TGC,TGC,AAC

23. ATGALDHSS-23: Tm=54, 60mer

AGG,AGA,CTC,TCT,CTG,AGG,TCG,AAG,CCA,TCC,TCG,AAC,CAG,TCA,AGA,ACC,TCA,CCT,GGC,CAT

24. ATGALDHSS-24: Tm=54, 40mer

TTA,GTT,CTG,GTG,GAT,TCC,AGA,TGG,CCA,GGT,GAG,GTT,CTT,G

***AtGLDHS:*** ATGACTAAGATCGAACTCAGAGCACTTGGAAACACTGGACTCAAGGTTTCTGCTGTCGGATTCGGTGCATCTCCACTCGGATCTGTCTTCGGACCAGTTGCTGAAGACGACGCTGTTGCAACTGTCAGAGAGGCTTTCAGACTCGGTATCAACTTCTTCGACACCTCTCCATACTACGGAGGAACCCTCTCTGAGAAGATGCTCGGTAAGGGACTCAAGGCTCTCCAAGTTCCTAGATCCGACTACATCGTTGCTACCAAGTGTGGTAGATACAAAGAGGGTTTCGACTTCTCTGCTGAGAGAGTCAGAAAGTCCATCGACGAGTCCTTGGAGAGACTCCAGCTCGACTACGTTGACATCCTCCACTGCCACGACATCGAGTTTGGATCTCTCGACCAGATCGTCTCTGAGACCATCCCTGCTCTCCAGAAGCTCAAGCAGGAGGGTAAGACCAGATTCATCGGTATCACTGGTCTCCCATTGGACATCTTCACCTACGTTCTCGACAGAGTTCCACCAGGAACTGTTGACGTTATCTTGTCTTACTGTCACTACGGTGTCAACGACTCTACCTTGCTCGACTTGCTCCCATACTTGAAGTCTAAGGGTGTTGGTGTCATCTCTGCTTCTCCATTGGCAATGGGACTCCTCACCGAACAAGGACCACCTGAGTGGCACCCTGCTTCTCCTGAACTCAAGTCCGCATCCAAGGCTGCTGTTGCTCACTGCAAGTCTAAGGGTAAGAAGATCACCAAGTTGGCTCTCCAGTACTCTCTCGCAAACAAGGAAATCTCTTCTGTCTTGGTTGGAATGTCTTCTGTCTCTCAGGTTGAAGAGAACGTTGCAGCAGTCACCGAGCTTGAGTCTCTCGGTATGGACCAGGAGACTCTCTCTGAGGTCGAAGCCATCCTCGAACCAGTCAAGAACCTCACCTGGCCATCTGGAATCCACCAGAACTAA

1. ATGLDHS-1: Tm=54, 60mer

ATG,ACT,AAG,ATC,GAA,CTC,AGA,GCA,CTT,GGA,AAC,ACT,GGA,CTC,AAG,GTT,TCT,GCT,GTC,GGA

2. ATGLDHS-2: Tm=54, 60mer

CAA,CTG,GTC,CGA,AGA,CAG,ATC,CGA,GTG,GAG,ATG,CAC,CGA,ATC,CGA,CAG,CAG,AAA,CCT,TGA

3. ATGLDHS-3: Tm=54, 60mer

ATC,TGT,CTT,CGG,ACC,AGT,TGC,TGA,AGA,CGA,CGC,TGT,TGC,AAC,TGT,CAG,AGA,GGC,TTT,CAG

4. ATGLDHS-4: Tm=54, 60mer

TCC,GTA,GTA,TGG,AGA,GGT,GTC,GAA,GAA,GTT,GAT,ACC,GAG,TCT,GAA,AGC,CTC,TCT,GAC,AGT

5. ATGLDHS-5: Tm=54, 60mer

ACA,CCT,CTC,CAT,ACT,ACG,GAG,GAA,CCC,TCT,CTG,AGA,AGA,TGC,TCG,GTA,AGG,GAC,TCA,AGG

6. ATGLDHS-6: Tm=54, 60mer

TTG,GTA,GCA,ACG,ATG,TAG,TCG,GAT,CTA,GGA,ACT,TGG,AGA,GCC,TTG,AGT,CCC,TTA,CCG,AGC

7. ATGLDHS-7: Tm=54, 60mer

GAC,TAC,ATC,GTT,GCT,ACC,AAG,TGT,GGT,AGA,TAC,AAA,GAG,GGT,TTC,GAC,TTC,TCT,GCT,GAG

8. ATGLDHS-8: Tm=54, 60mer

GGA,GTC,TCT,CCA,AGG,ACT,CGT,CGA,TGG,ACT,TTC,TGA,CTC,TCT,CAG,CAG,AGA,AGT,CGA,AAC

9. ATGLDHS-9: Tm=54, 60mer

CGA,GTC,CTT,GGA,GAG,ACT,CCA,GCT,CGA,CTA,CGT,TGA,CAT,CCT,CCA,CTG,CCA,CGA,CAT,CGA

10. ATGLDHS-10: Tm=54, 60mer

AGG,GAT,GGT,CTC,AGA,GAC,GAT,CTG,GTC,GAG,AGA,TCC,AAA,CTC,GAT,GTC,GTG,GCA,GTG,GAG

11. ATGLDHS-11: Tm=54, 60mer

TCG,TCT,CTG,AGA,CCA,TCC,CTG,CTC,TCC,AGA,AGC,TCA,AGC,AGG,AGG,GTA,AGA,CCA,GAT,TCA

12. ATGLDHS-12: Tm=54, 60mer

ACG,TAG,GTG,AAG,ATG,TCC,AAT,GGG,AGA,CCA,GTG,ATA,CCG,ATG,AAT,CTG,GTC,TTA,CCC,TCC

13. ATGLDHS-13: Tm=54, 60mer

TTG,GAC,ATC,TTC,ACC,TAC,GTT,CTC,GAC,AGA,GTT,CCA,CCA,GGA,ACT,GTT,GAC,GTT,ATC,TTG

14. ATGLDHS-14: Tm=54, 60mer

CGA,GCA,AGG,TAG,AGT,CGT,TGA,CAC,CGT,AGT,GAC,AGT,AAG,ACA,AGA,TAA,CGT,CAA,CAG,TTC

15. ATGLDHS-15: Tm=54, 60mer

CAA,CGA,CTC,TAC,CTT,GCT,CGA,CTT,GCT,CCC,ATA,CTT,GAA,GTC,TAA,GGG,TGT,TGG,TGT,CAT

16. ATGLDHS-16: Tm=54, 60mer

TTG,TTC,GGT,GAG,GAG,TCC,CAT,TGC,CAA,TGG,AGA,AGC,AGA,GAT,GAC,ACC,AAC,ACC,CTT,AGA

17. ATGLDHS-17: Tm=54, 60mer

TGG,GAC,TCC,TCA,CCG,AAC,AAG,GAC,CAC,CTG,AGT,GGC,ACC,CTG,CTT,CTC,CTG,AAC,TCA,AGT

18. ATGLDHS-18: Tm=54, 60mer

CCC,TTA,GAC,TTG,CAG,TGA,GCA,ACA,GCA,GCC,TTG,GAT,GCG,GAC,TTG,AGT,TCA,GGA,GAA,GCA

19. ATGLDHS-19: Tm=54, 60mer

GCT,CAC,TGC,AAG,TCT,AAG,GGT,AAG,AAG,ATC,ACC,AAG,TTG,GCT,CTC,CAG,TAC,TCT,CTC,GCA

20. ATGLDHS-20: Tm=54, 60mer

CAG,AAG,ACA,TTC,CAA,CCA,AGA,CAG,AAG,AGA,TTT,CCT,TGT,TTG,CGA,GAG,AGT,ACT,GGA,GAG

21. ATGLDHS-21: Tm=54, 60mer

CTT,GGT,TGG,AAT,GTC,TTC,TGT,CTC,TCA,GGT,TGA,AGA,GAA,CGT,TGC,AGC,AGT,CAC,CGA,GCT

22. ATGLDHS-22: Tm=54, 60mer

GAC,CTC,AGA,GAG,AGT,CTC,CTG,GTC,CAT,ACC,GAG,AGA,CTC,AAG,CTC,GGT,GAC,TGC,TGC,AAC

23. ATGLDHS-23: Tm=54, 60mer

AGG,AGA,CTC,TCT,CTG,AGG,TCG,AAG,CCA,TCC,TCG,AAC,CAG,TCA,AGA,ACC,TCA,CCT,GGC,CAT

24. ATGLDHS-24: Tm=54, 40mer

TTA,GTT,CTG,GTG,GAT,TCC,AGA,TGG,CCA,GGT,GAG,GTT,CTT,G

**Notes 2:** Detailed procedures of Proteomics assay

**Protein Extraction**

Cell samples treated under different conditions were sonicated three times on ice using a high intensity ultrasonic processor (Scientz) in lysis buffer (8 M urea, 1% Protease Inhibitor Cocktail). The remaining debris was removed by centrifugation at 12,000g at 4 °C for 10 min. Finally, the supernatant was collected and the protein concentration was determined with BCA kit according to the manufacturer’s instructions.

**Trypsin Digestion**

For digestion, the protein solution was reduced with 5 mM dithiothreitol for 30 min at 56 °C and alkylated with 11 mM iodoacetamide for 15 min at room temperature in darkness. The protein sample was then diluted by adding 100 mM TEAB to urea concentration less than 2M. Finally, trypsin was added at 1:50 trypsin-to-protein mass ratio for the first digestion overnight and 1:100 trypsin-to-protein mass ratio for a second 4 h-digestion.

**TMT/iTRAQ Labeling**

After trypsin digestion, peptide was desalted by Strata X C18 SPE column (Phenomenex) and vacuum-dried. Peptide was reconstituted in 0.5 M TEAB and processed according to the manufacturer’s protocol for TMT kit/iTRAQ kit. Briefly, one unit of TMT/iTRAQ reagent were thawed and reconstituted in acetonitrile. The peptide mixtures were then incubated for 2 h at room temperature and pooled, desalted and dried by vacuum centrifugation.

**HPLC Fractionation**

The tryptic peptides were fractionated into fractions by high pH reverse-phase HPLC using Agilent 300Extend C18 column (5 μm particles, 4.6 mm ID, 250 mm length). Briefly, peptides were first separated with a gradient of 8% to 32% acetonitrile (pH 9.0) over 60 min into 60 fractions. Then, the peptides were combined into 18 fractions and dried by vacuum centrifuging.

**LC-MS/MS Analysis**

The tryptic peptides were dissolved in 0.1% formic acid (solvent A), directly loaded onto a home-made reversed-phase analytical column (15-cm length, 75 μm i.d.). The gradient was comprised of an increase from 6% to 23% solvent B (0.1% formic acid in 98% acetonitrile) over 26 min, 23% to 35% in 8 min and climbing to 80% in 3 min then holding at 80% for the last 3 min, all at a constant flow rate of 400 nL/min on an EASY-nLC 1000 UPLC system.

The peptides were subjected to NSI source followed by tandem mass spectrometry (MS/MS) in Q Exactive^TM^ Plus (Thermo) coupled online to the UPLC. The electrospray voltage applied was 2.0 kV. The m/z scan range was 350 to 1800 for full scan, and intact peptides were detected in the Orbitrap at a resolution of 70,000. Peptides were then selected for MS/MS using NCE setting as 28 and the fragments were detected in the Orbitrap at a resolution of 17,500. A data-dependent procedure that alternated between one MS scan followed by 20 MS/MS scans with 15.0s dynamic exclusion. Automatic gain control (AGC) was set at 5E4. Fixed first mass was set as 100 m/z.

**Database Search**

The resulting MS/MS data were processed using Maxquant search engine (v.1.5.2.8). Tandem mass spectra were searched against *Escherichia coli* K-12 MG 1655 database concatenated with exogenous nine phenol degradation proteins sequences and reverse decoy database. Trypsin/P was specified as cleavage enzyme allowing up to 2 missing cleavages. The mass tolerance for precursor ions was set as 20 ppm in First search and 5 ppm in Main search,and the mass tolerance for fragment ions was set as 0.02 Da. Carbamidomethyl on Cys was specified as fixed modification and oxidation on Met was specified as variable modifications. FDR was adjusted to < 1% and minimum score for peptides was set > 40.
